# Targeting RUNX1 protects against diastolic dysfunction in a two-hit mouse model of heart failure with preserved ejection fraction

**DOI:** 10.1093/cvr/cvag106

**Published:** 2026-05-26

**Authors:** Ali Ali Mohamed Elbassioni, Anmar A Raheem, Jian Song, Alexander S Johnston, Cara Trivett, Hong Lin, Haobo Zhang, Ashley Bradley, Erin Higgins, Cameron R Thomson, Leanne Mooney, Yen Chin Koay, Dylan O’Toole, Pawel Herzyk, Colin Nixon, Karen Blyth, Mark Hughes, Nawwar Al-Attar, John F O’Sullivan, Ninian N Lang, Colin Berry, Thomas Braun, Gabriele G Schiattarella, Mauro Giacca, Martin W McBride, Stuart A Nicklin, Ewan R Cameron, Christopher M Loughrey, Eilidh A MacDonald

**Affiliations:** School of Cardiovascular and Metabolic Health, University of Glasgow, Glasgow, UK; Department of Cardiothoracic Surgery, Suez Canal University, Ismailia, Egypt; School of Cardiovascular and Metabolic Health, University of Glasgow, Glasgow, UK; School of Cardiovascular and Metabolic Health, University of Glasgow, Glasgow, UK; School of Cardiovascular and Metabolic Health, University of Glasgow, Glasgow, UK; School of Cardiovascular and Metabolic Health, University of Glasgow, Glasgow, UK; School of Cardiovascular and Metabolic Health, University of Glasgow, Glasgow, UK; School of Cardiovascular and Metabolic Health, University of Glasgow, Glasgow, UK; School of Cardiovascular and Metabolic Health, University of Glasgow, Glasgow, UK; School of Cardiovascular and Metabolic Health, University of Glasgow, Glasgow, UK; School of Cardiovascular and Metabolic Health, University of Glasgow, Glasgow, UK; School of Cardiovascular and Metabolic Health, University of Glasgow, Glasgow, UK; School of Medical Sciences, The University of Sydney, Sydney, New South Wales, Australia; School of Cardiovascular and Metabolic Health, University of Glasgow, Glasgow, UK; School of Molecular Biosciences, University of Glasgow, Glasgow, UK; Shared Research Facility, University of Glasgow, Glasgow, UK; Cancer Research UK Scotland Institute, Glasgow, UK; Cancer Research UK Scotland Institute, Glasgow, UK; School of Cancer Sciences, University of Glasgow, Glasgow, UK; Cancer Research UK Scotland Institute, Glasgow, UK; School of Cardiovascular and Metabolic Health, University of Glasgow, Glasgow, UK; School of Medical Sciences, The University of Sydney, Sydney, New South Wales, Australia; Cardiometabolic Medicine, The University of Sydney, Sydney, New South Wales, Australia; Department of Cardiology, Royal Prince Alfred Hospital, Camperdown, New South Wales, Australia; The Baird Institute for Applied Heart and Lung Surgical Research, Sydney, New South Wales, Australia; School of Cardiovascular and Metabolic Health, University of Glasgow, Glasgow, UK; School of Cardiovascular and Metabolic Health, University of Glasgow, Glasgow, UK; Department of Cardiac Development and Remodelling, Max Planck Institute for Heart and Lung Research, Bad Nauheim, Germany; Deutsches Herzzentrum der Charité, Department of Cardiology, Angiology and Intensive Care Medicine, Max Rubner Center for Cardiovascular Metabolic Renal Research (MRC), Charité-Universitätsmedizin Berlin, Germany, Berlin, Germany; DZHK (German Centre for Cardiovascular Research), Partner Site Berlin, Berlin, Germany; Translational Approaches in Heart Failure and Cardiometabolic Disease, Max Delbrück Center for Molecular Medicine in the Helmholtz Association (MDC), Berlin, Germany; Friede Springer Cardiovascular Prevention Center at Charité – Universitätsmedizin Berlin, Germany; Experimental and Clinical Research Center (ECRC), a Cooperation of Charité-Universitätsmedizin Berlin and Max Delbruck Center for Molecular Medicine (MDC); Division of Cardiology, Department of Advanced Biomedical Sciences, Federico II University, Naples, Italy; Department of Cardiothoracic Surgery, Suez Canal University, Ismailia, Egypt; School of Cardiovascular and Metabolic Health, University of Glasgow, Glasgow, UK; School of Cardiovascular and Metabolic Health, University of Glasgow, Glasgow, UK; School of Biodiversity One Health and Veterinary Medicine, University of Glasgow, Glasgow, UK; School of Cardiovascular and Metabolic Health, University of Glasgow, Glasgow, UK; School of Biodiversity One Health and Veterinary Medicine, University of Glasgow, Glasgow, UK; School of Cardiovascular and Metabolic Health, University of Glasgow, Glasgow, UK

**Keywords:** Heart failure with preserved ejection fraction, Metabolic heart failure, Diastolic dysfunction, Hypertrophy, Pulmonary oedema, Exercise intolerance

## Abstract

**Aims:**

Heart failure with preserved ejection fraction (HFpEF) continues to increase in prevalence and has limited treatment options. HFpEF is a systemic condition with a broad phenotype including diastolic dysfunction, pulmonary oedema, exercise intolerance, and left ventricular hypertrophy, collectively resulting in enhanced morbidity and mortality. The transcription factor RUNX1 has recently been identified as a mediator of pathological changes in multiple cardiac diseases; however, its role in HFpEF remained unknown.

**Methods and results:**

Here, we show that inhibition of *Runx*1 limits adverse cardiac remodelling in a mouse model of HFpEF. Cardiomyocyte-specific tamoxifen-inducible *Runx*1-deficient mice with HFpEF are protected, with preservation of diastolic function, and attenuation of pulmonary oedema, exercise intolerance, and hypertrophy. Furthermore, targeting *Runx*1 in HFpEF by using gene transfer or small-molecule inhibitor Ro5-3335 improves diastolic function and reduces pulmonary oedema, both in female and male mice.

**Conclusion:**

Overall, this work enhances our understanding of RUNX1 in cardiac disease and presents a novel translational target for the treatment of HFpEF.

## Graphical Abstract

**Graphical Abstract:**
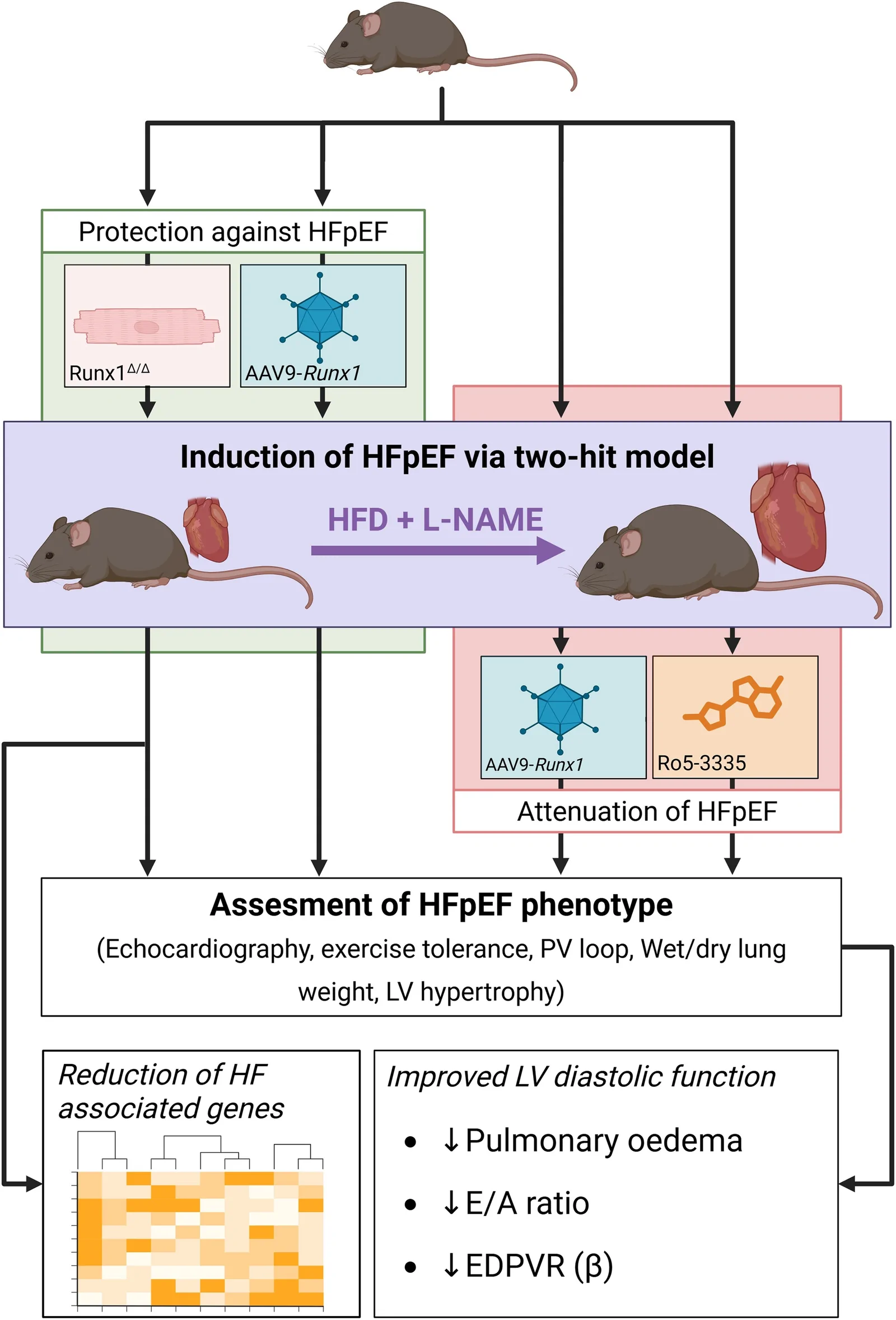

**Time for primary review: 84 days**

**See the editorial comment for this article ‘RUNX1: a new beat in the heart of diastolic dysfunction’, by M.H. Elbatreek and D.J. Lefer, https://doi.org/10. 1093/cvr/cvag097.**

## 1. Introduction

Heart failure (HF), a complex syndrome in which the heart is unable to meet the metabolic demands of the body, leads to considerable morbidity and mortality worldwide. It is classically categorized by the proportion of blood ejected from the left ventricle (LV) with each beat, the ejection fraction (EF). HF with reduced EF (HFrEF) has been heavily investigated for many years and there are several treatment options available that reduce mortality.^1^ More elusive; however, is HF with preserved EF (HFpEF) which is increasing in prevalence and is poorly understood.^2^ Despite multiple advances in the treatment of HFrEF, these treatments are not effective for use in HFpEF, resulting in limited therapeutic options.^3^ HFpEF is a multi-morbidity syndrome, often developing alongside hypertension, metabolic stress, and diabetes, and results in diastolic dysfunction, pulmonary oedema, hypertrophy, and exercise intolerance.^3^ HFpEF includes a wide range of clinical phenotypes and pathophysiological heterogeneity.^4^ Elucidating molecular or cellular factors contributing to the development of HFpEF is essential and an important step towards identifying therapeutic targets.

The master regulator transcription factor, RUNX1, is minimally expressed in the adult heart but can be reactivated in the context of cardiac pathology. Using pre-clinical animal model systems, it has been shown to be a mediator and therapeutic target against adverse cardiac remodelling following myocardial infarction (MI)—a major cause of HFrEF,^5–7^ and in a transaortic constriction HFrEF model.^8,9^ Targeting *Runx*1 in the post-MI heart results in improved systolic function, calcium handling, and preservation of genes involved in oxidative phosphorylation.^5,6,10^ Large scale analysis of RNAseq studies on human myocardium^11–17^ demonstrates that *Runx*1 expression is increased in several cardiac pathologies including MI, hypertrophic cardiomyopathy, and dilated cardiomyopathy (see Supplementary material online, *Figure S1* and *Table S1*). Given its established role as a maladaptive regulator in diverse forms of cardiac pathologies, we hypothesized a potential role for RUNX1 in the pathophysiology of HFpEF.^18^ The aim of this study was to use a pre-clinical model of HFpEF to interrogate the role of RUNX1 in the development of HFpEF and to identify its potential as a therapeutic target for the treatment of HFpEF.

## 2. Methods

Detailed methods and statistical analysis are presented in the Supplementary material online, *Methods*. We used a previously established,^19^ two-hit model (2HM) that combines administration of a high-fat diet (HFD) and inhibition of nitric oxide synthase with N^ω^-nitro-L-arginine methyl ester (L-NAME) in drinking water to induce a HFpEF phenotype and compared changes to age-matched controls (CTRL) fed a regular chow diet and normal drinking water.^19^ We utilized cardiomyocyte-specific tamoxifen-inducible *Runx1*-deficient (*Runx1*^Δ/Δ^) mice and floxed genetic-control mice (*Runx1*^fl/fl^ original line from Growney *et al*.^20^) generated as previously described to reduce *Runx*1 expression after being given a single intraperitoneal injection of 40 mg/kg tamoxifen.^21^ RUNX1 was also targeted by either adeno-associated virus (AAV)-mediated delivery of shRNA (see Supplementary material online, *Figure S2*) or a small-molecule inhibitor of RUNX1, Ro5-3335, as detailed in the Supplementary material online, *Methods*. Bulk RNA sequencing and subsequent pathway analysis was performed on LV tissue samples from *Runx1*^fl/fl^ and *Runx1*^Δ/Δ^ mice at baseline and after the 2HM protocol. Using prior biological knowledge from the Ingenuity knowledge base, the cardiotoxicity networks and functional analyses were generated using QIAGEN Ingenuity Pathway Analysis (IPA) (https://www.qiagenbioinformatics.com/products/ingenuity-pathway-analysis/). The focus on cardiotoxicity processes considers the likely activation and inhibition of biological processes between the two strain comparisons (2HM-*Runx1*^fl/fl^ compared with CTRL-*Runx1*^fl/fl^ and 2HM-*Runx1*^Δ/Δ^ and CTRL-*Runx1*^Δ/Δ^) using a *Z*-score. Differences in *Z*-scores were identified.

## 3. Results

### 3.1 Runx1 expression in HFpEF

To investigate the involvement of RUNX1 in the pathophysiology of HFpEF, we measured *Runx*1 gene expression in cardiomyocytes from our 2HM in C57BL/6N mice. *Runx1* expression was significantly elevated in 2HM mice compared with CTRL (see Supplementary material online, *Figure S2*) suggesting that Runx1 has potential to drive adverse cardiac remodelling in HFpEF, as is the case post-MI.

### 3.2 Effect of cardiomyocyte-specific *Runx1* deficiency on the development of HFpEF

**Figure 1:**
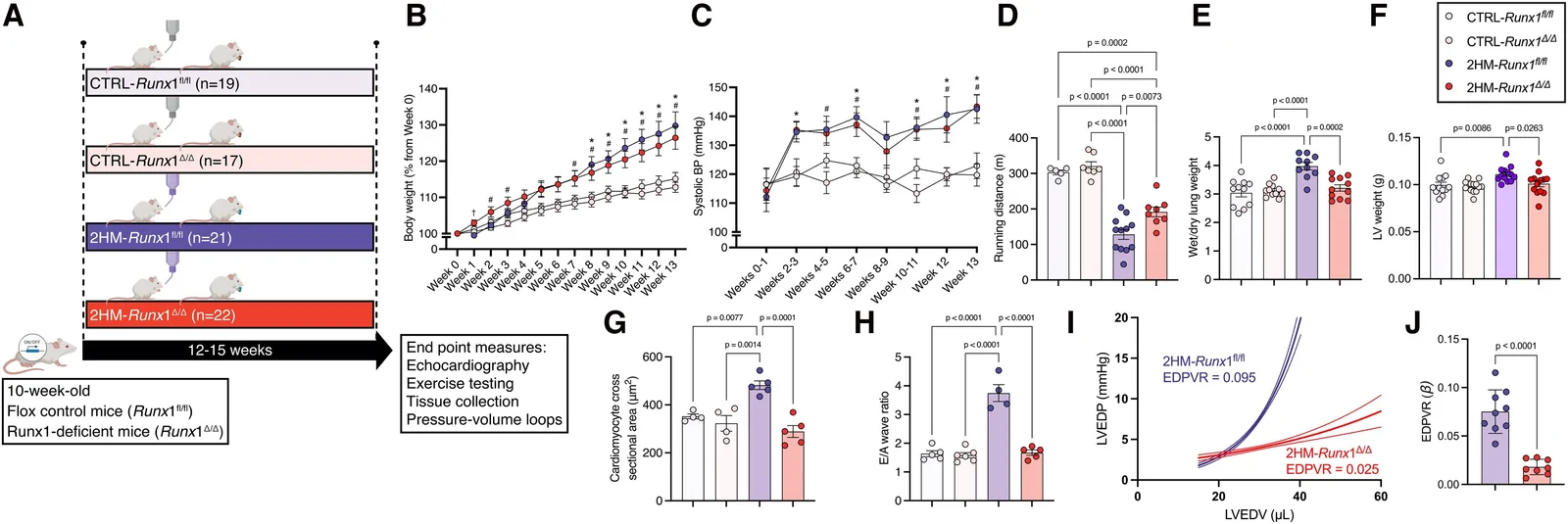
*Runx*1-deficient mice are protected against HFpEF phenotype. (*A*) Schematic of two-hit protocol and experimental groups, created with BioRender. (*B*) Body weight over the experimental protocol in each of the groups. *P < 0.05 for 2HM-*Runx*1^fl/fl^ (*n* = 21) compared with CTRL-*Runx*1^fl/fl^ (*n* = 19), ^#^*P* < 0.05 for 2HM-*Runx*1^Δ/Δ^ (*n* = 22) compared with CTRL-*Runx*1^Δ/Δ^ (*n* = 17), and ^†^*P* < 0.05 for 2HM-*Runx*1^Δ/Δ^ compared with 2HM-*Runx*1^fl/fl^ by mixed-effects analysis. (*C*) SBP over the experimental protocol. *P < 0.05 for 2HM-*Runx*1^fl/fl^ (*n* = 25) compared with CTRL-*Runx*1^fl/fl^ (*n* = 18) and ^#^*P* < 0.05 for 2HM-*Runx*1^Δ/Δ^ (*n* = 19) compared with CTRL- *Runx*1^Δ/Δ^ (*n* = 25) by mixed-effects analysis. Characterization of the HFpEF phenotype: (*D*) exercise intolerance was quantified by running distance (CTRL-*Runx1*^fl/fl^: *n* = 5; CTRL- *Runx1*^Δ/Δ^: *n* = 7; 2-HM-*Runx1*^fl/fl^: *n* = 12; 2-HM-*Runx1*^Δ/Δ^: *n* = 8) by mixed-effects analysis. (*E*) Pulmonary oedema quantified by wet to dry lung weight ratio (CTRL-*Runx1*^fl/fl^: *n* = 10; CTRL-*Runx1*^Δ/Δ^: *n* = 11; 2-HM-*Runx1*^fl/fl^: *n* = 11; 2-HM-*Runx1*^Δ/Δ^: *n* = 11) by two-way ANOVA (Analysis of Variance). Hypertrophy was quantified by (*F*) LV weight (CTRL-*Runx1*^fl/fl^: *n* = 12; CTRL- *Runx1*^Δ/Δ^: *n* = 13; 2-HM-*Runx1*^fl/fl^: *n* = 12; 2-HM-*Runx1*^Δ/Δ^: *n* = 13) by two-way ANOVA and by (*G*) cardiomyocyte cross-sectional area assessed following wheat germ agglutinin staining (CTRL-*Runx1*^fl/fl^: *n* = 4; CTRL-*Runx1*^Δ/Δ^: *n* = 4; 2-HM-*Runx1*^fl/fl^: *n* = 5; 2-HM-*Runx1*^Δ/Δ^: *n* = 5) by two-way ANOVA. Diastolic function quantified by (*H*) *E*/*A* wave ratio from pulsed wave Doppler echocardiography (CTRL-*Runx1*^fl/fl^: *n* = 5; CTRL-*Runx1*^Δ/Δ^: *n* = 6; 2-HM-*Runx1*^fl/fl^; *n* = 4, 2-HM-*Runx1*^Δ/Δ^; *n* = 5) by two-way ANOVA and by the slope (β) of the EDPVR derived from the exponential equation: Left ventricular end diastolic pressure (LVEDP) = curve fitting constant × *e*^[stiffness constant^  ^×^  ^LV end-diastolic volume]^. (*I*) Representative curves (error lines denote 95% confidence interval); and (*J*) data set (2HM-*Runx1*^fl/fl^: *n* = 9; 2HM-*Runx1*^Δ/Δ^: *n* = 8) by *t*-test.

To evaluate the functional contribution of Runx1, we utilized male *Runx1*^Δ/Δ^ mice and floxed control mice (*Runx1*^fl/fl^) on the 2HM and CTRL protocols compared with age-matched genetic controls (CTRL-*Runx1*^fl/fl^: *n* = 19, CTRL-*Runx1*^Δ/Δ^: *n* = 17, 2HM-*Runx1*^fl/fl^: *n* = 21, 2HM-*Runx1*^Δ/Δ^: *n* = 22, *Figure 1A*). Each of the two-hits were observed in 2HM-*Runx1*^fl/fl^ and 2HM-*Runx1*^Δ/Δ^ male mice with an increase in body weight (8.0 ± 0.9 and 7.0 ± 0.9 g, respectively both *P* < 0.05; *Figure 1B*) and systolic blood pressure (SBP, 22 ± 10 and 20 ± 6 mmHg, respectively, both *P* < 0.05; *Figure 1C*) over the course of the protocol compared with respective CTRL groups. We confirmed that neither 2HM group had developed HFrEF by assessing whole heart contractile function as measured by fractional shortening *via* echocardiography and EF from pressure–volume catheters (see Supplementary material online, *Figure S3A*).

We then evaluated other key features of the HFpEF phenotype. At the end of the protocol, 2HM-*Runx1*^fl/fl^ mice had developed exercise intolerance, demonstrated by a reduction in running distance compared with CTRL-*Runx1*^fl/fl^ mice (128 ± 14 vs. 296 ± 18 m, respectively, *P* < 0.05; *Figure 1D*). 2HM-*Runx1*^Δ/Δ^ mice also had a reduction in running distance compared with their genetic control (2HM-*Runx1*^Δ/Δ^: 192 ± 14 m, CTRL-*Runx1*^Δ/Δ^: 327 ± 22 m, *P* < 0.05; *Figure 1D*); however, the exercise intolerance was attenuated because 2HM-*Runx1*^Δ/Δ^ mice ran greater distances than 2HM-*Runx1*^fl/fl^ mice (128 ± 14 vs. 192 ± 14 m, *P* < 0.05; *Figure 1D*). *Runx*1 deficiency also protected mice from developing pulmonary oedema as evaluated by the wet to dry lung weight ratio, which was increased in 2HM-*Runx1*^fl/fl^ compared with in CTRL-*Runx1*^fl/fl^ mice (3.90 ± 0.2 vs. 2.84 ± 0.2, *P* < 0.05; *Figure 1E*). Conversely, there was no difference between 2HM-*Runx1*^Δ/Δ^ and CTRL-*Runx1*^Δ/Δ^ mice (3.14 ± 0.2 vs. 3.00 ± 0.1; *Figure 1E*), and 2HM-*Runx1*^Δ/Δ^ mice had significantly lower wet to dry lung weight ratio than 2HM-*Runx1*^fl/fl^ mice (3.14 ± 0.2 vs. 3.90 ± 0.2, *P* < 0.05; *Figure 1E*). *Runx*1 deficiency was also protective against development of hypertrophy as measured by LV weight. 2HM-*Runx1*^fl/fl^ had increased LV weight compared with CTRL-*Runx1*^fl/fl^ mice (0.111 g ± 0.002 vs. 0.100 g ± 0.003, *P* < 0.05; *Figure 1F*) whereas 2HM-*Runx1*^Δ/Δ^ mice did not have an increase in LV weight compared with CTRL-*Runx1*^Δ/Δ^ mice (0.101 ± 0.003 vs. 0.098 ± 0.002, *P* > 0.05; *Figure 1F*). An additional indicator, relevant to concentric hypertrophy, is cardiomyocyte cross-sectional area. In contrast to 2HM-*Runx1*^fl/fl^ animals, 2HM-*Runx1*^Δ/Δ^ mice showed no increase in cardiomyocyte cross-sectional area compared with their relative control group (CTRL-*Runx1*^fl/fl^: 352 ± 9.9 μm2, CTRL-*Runx1*^Δ/Δ^: 323 ± 32.4 μm2, 2HM-*Runx1*^fl/fl^: 482 ± 18.0 μm2, 2HM-*Runx1*^Δ/Δ^: 289 ± 24.8 μm2; *Figure 1G*; Supplementary material online, *Figure S3B*). Further, posterior and anterior wall thickness measured during systole with M-mode echocardiography was increased in the 2HM-*Runx1*^fl/fl^ mice but not in the 2HM-*Runx1*^Δ/Δ^ mice, compared with relevant controls (see Supplementary material online, *Figure S3C*). In addition to hypertrophy, there was a striking preservation of diastolic function in cardiomyocyte-specific *Runx*1-deficient mice, quantified by *E*/*A* wave ratio from pulsed wave Doppler echocardiography. Compared with CTRL-*Runx1*^fl/fl^ and CTRL-*Runx1*^Δ/Δ^, 2HM-*Runx1*^fl/fl^ had a higher *E*/*A* ratio whereas the *E*/*A* ratio in 2HM-*Runx1*^Δ/Δ^ mice was not different from either control group (CTRL-*Runx1*^fl/fl^: 1.63 ± 0.10, CTRL-*Runx1*^Δ/Δ^: 1.58 ± 0.09, 2HM-*Runx1*^fl/fl^: 3.75 ± 0.29, 2HM-*Runx1*^Δ/Δ^: 1.67 ± 0.09; *Figure 1H*; Supplementary material online, *Figure S3D*). An independent measure of LV chamber stiffness was calculated by fitting the slope of the load-independent end-diastolic pressure–volume relationship (EDPVR) measured using intracardiac pressure–volume catheters. 2HM-*Runx1*^fl/fl^ mice had a steeper EDPVR slope than 2HM-*Runx1*^Δ/Δ^ mice, indicating better diastolic function in the 2HM-*Runx1*^Δ/Δ^ mice (0.075 ± 0.008 vs. 0.018 ± 0.003, *P* < 0.05; *Figure 1I* and *J*; Supplementary material online, *Figure S3E*). Peripheral organs were also collected to investigate systemic effects of cardiomyocyte-specific *Runx*1deficiency. Liver, right kidney, and left kidney weights [all normalized to tibial length (TL)] were increased in 2HM-*Runx1*^fl/fl^ compared with CTRL-*Runx1*^fl/fl^ mice but were not different between *Runx1*^Δ/Δ^ mouse groups (see Supplementary material online, *Figure S3F*).

### 3.3 *Runx1* RNA interference using AAV serotype 9 (AAV9) attenuates diastolic dysfunction in HFpEF

**Figure 2:**
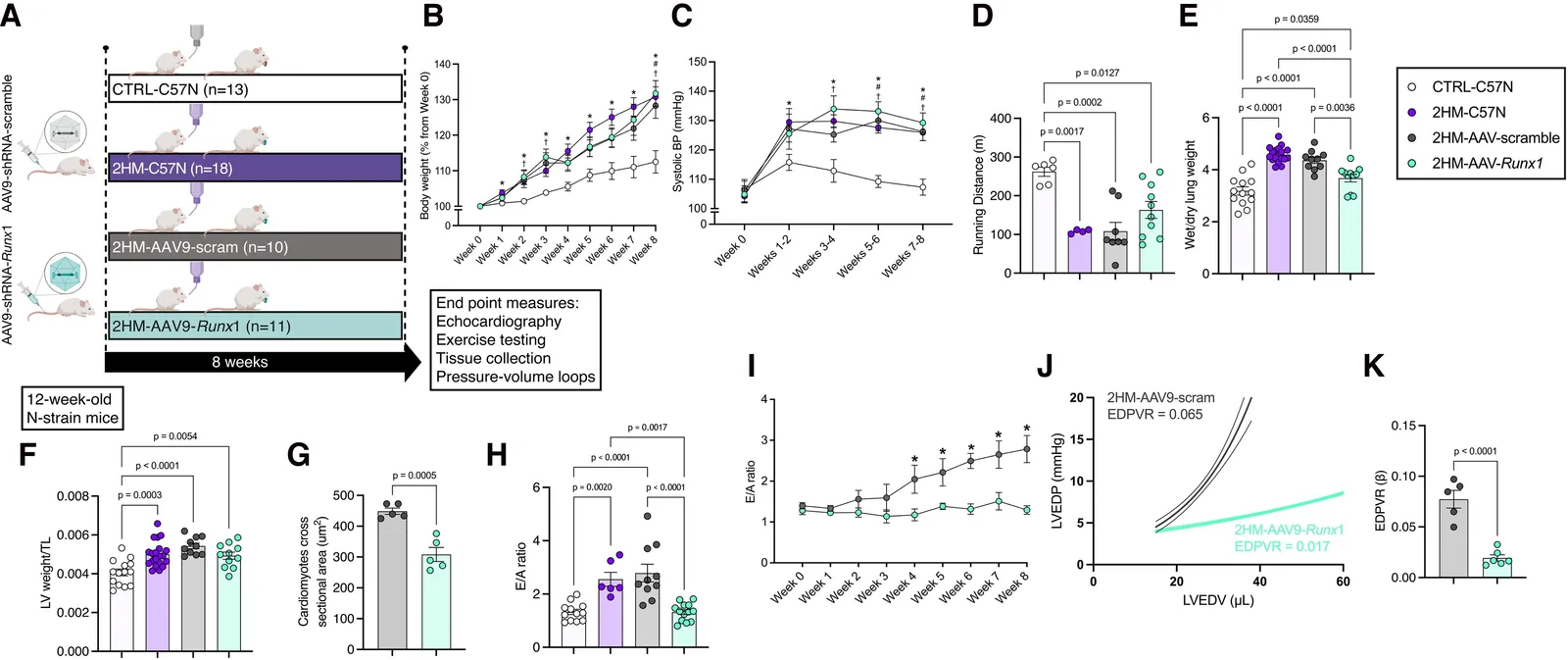
AAV9-mediated knockdown of *Runx*1 protects against diastolic dysfunction. (*A*) Schematic of two-hit protocol and experimental groups, created with BioRender. (*B*) Body weight over the experimental protocol. *P < 0.05 for 2HM-C57N (*n* = 18) compared with CTRL-C57N (*n* = 13), ^#^*P* < 0.05 for 2HM-AAV9-scram (*n* = 10) compared with CTRL-C57N, and ^†^*P* < 0.05 for 2HM- AAV9-*Runx*1 (*n* = 11) compared with CTRL-C57N by mixed-effects analysis. (*C*) SBP over the experimental protocol. *P < 0.05 for 2HM-C57N (*n* = 38) compared with CTRL-C57N (*n* = 31), ^#^*P* < 0.05 for 2HM-AAV9-scram (*n* = 10) compared with CTRL-C57N, and ^†^*P* < 0.05 for 2HM-AAV9-*Runx*1 (*n* = 10) compared with CTRL-C57N by mixed-effects analysis. Characterization of the HFpEF phenotype: (*D*) exercise intolerance was quantified by running distance (CTRL-C57N: *n* = 6; 2HM-C57N: *n* = 4; 2HM-AAV9-scram: *n* = 8; 2HM-AAV9-*Runx*1: *n* = 10) by one-way ANOVA. (*E*) Pulmonary oedema quantified by wet to dry lung weight ratio (CTRL-C57N: *n* = 13; 2HM-C57N: *n* = 13; 2HM-AAV9-scram: *n* = 10; 2HM-AAV9-*Runx*1: *n* = 11) by one-way ANOVA. Hypertrophy was quantified by (*F*) LV weight normalized to TL (CTRL-C57N: *n* = 14; 2HM-C57N: *n* = 20; 2HM-AAV9-scram: *n* = 10; 2HM-AAV9-*Runx*1: *n* = 11) by one-way ANOVA and by (*G*) cardiomyocyte cross-sectional area assessed following wheat germ agglutinin staining (2HM-AAV9-scram: *n* = 5; 2HM-AAV9-*Runx*1: *n* = 5) by *t*-test. Diastolic function quantified by *E*/*A* wave ratio from pulsed wave Doppler echocardiography (CTRL-C57N: *n* = 12; 2HM-C57N: *n* = 6; 2HM-AAV9-scram: *n* = 9; 2HM-AAV9-*Runx*1: *n* = 13) on (*H*) the final week by one-way ANOVA and (*I*) over the protocol by two-way ANOVA; and by the slope (β) of the EDPVR derived from the exponential equation: (LVEDP = curve fitting constant × *e*^[stiffness constant^  ^×^  ^LV end-diastolic volume]^), 2HM-AAV9-scram: *n* = 5; 2HM-AAV9-*Runx*1: *n* = 6. (*J*) Representative curves (error lines denote 95% confidence interval); and (*K*) data set by *t*-test.

Given the striking phenotypic differences observed in 2HM-*Runx1*^Δ/Δ^ mice compared with 2HM-*Runx1*^fl/fl^, we next sought to target RUNX1 using additional approaches to determine whether *Runx1-*deficiency could prevent the development of HFpEF. To do this we utilized a viral vector-mediated gene delivery approach with AAV9-*Runx*1-shRNA to knockdown *Runx*1 in our 2HM of HFpEF, which we previously validated.^21^ We injected 12-week-old C57BL/6N male mice *via* the tail vein with AAV9-scramble-shRNA (2HM-AAV9-scram, *n* = 10) or AAV9-*Runx*1-shRNA (2HM-AAV9-*Runx*1, *n* = 11) after which mice were placed on the 2HM protocol for 8 weeks for comparison to age-matched C57BL/6N mice on the 2HM (2HM-C57N, *n* = 18) or control (CTRL-C57N, *n* = 13) protocols (*Figure 2A*). Once again, we confirmed the efficacy of our 2HM by measuring changes in body weight (*Figure 2B*), SBP (*Figure 2C*), and preservation of fractional shortening from echocardiography (see Supplementary material online, *Figure S4A*) over the duration of the protocol. At the end of the protocol, RNAScope analysis confirmed a significant reduction of *Runx1* expression in 2HM-AAV9-*Runx1* hearts (2HM-AAV9-*Runx*1, *n* = 6) compared with 2HM-AAV9-scramble-shRNA controls (2HM-AAV9-scram, *n* = 6) (see Supplementary material online, *Figure S4B*). Targeting *Runx*1 with AAV9-*Runx*1-shRNA was effective in preventing a number of the key features of the HFpEF phenotype. Exercise intolerance was observed in all three 2HM groups compared with CTRL-C57N, but with no difference in running distance between 2HM groups (CTRL-C57N: 262 ± 12 m, 2HM-C57N: 108 ± 3 m, 2HM-AAV9-scram: 108 ± 23 m, 2HM-AAV9-*Runx*1: 163 ± 22 m; *Figure 2D*). AAV9-*Runx*1 did, however, attenuate the development of pulmonary oedema compared with the other two 2HM groups, quantified by wet to dry lung weight ratio (CTRL-C57N: 3.18 ± 0.16, 2HM-C57N: 4.58 ± 0.07, 2HM-AAV9-scram: 4.36 ± 0.12, 2HM-AAV9-*Runx*1: 3.68 ± 0.15; *Figure 2E*). As with exercise testing, left ventricular weight normalised to tibial length (LV/TL) was increased in 2HM groups compared with CTRL but was not different between 2HM groups (CTRL-C57N: 4.1 ± 0.2 × 10^−3^, 2HM-C57N: 5.0 ± 0.2 × 10^−3^, 2HM-AAV9-scram: 5.4 ± 0.2 × 10^−3^, 2HM-AAV9-*Runx*1: 4.9 ± 0.2 × 10^−3^; *Figure 2F*). However, cardiomyocyte cross-sectional area of 2HM-AAV9-Runx1 was less than the 2HM-AAV9-scram group (2HM-AAV9-scram: 449 ± 10 μm2 vs. 2HM-AAV9-*Runx*1: 308 ± 23 μm2; *Figure 2G*; Supplementary material online, *Figure S4C*). Most striking was the preservation of diastolic function by targeting *Runx*1 with AAV9. E/A ratio was increased in both 2HM-C57N and 2HM-AAV9-scram groups compared with CTRL-C57N but was not increased in 2HM-AAV9-*Runx*1 compared with CTRL-C57N (CTRL-C57N: 1.32 ± 0.10, 2HM-C57N: 2.55 ± 0.26, 2HM-AAV9-scram: 2.78 ± 0.33, 2HM-AAV9-*Runx*1: 1.29 ± 0.12; *Figure 2H* and *I*; the latter figure demonstrating change over time, Supplementary material online, *Figure S4D*). This was also consistent with EDPVR, which was markedly lower in the 2HM-AAV9-*Runx*1 group compared with 2HM-AAV9-scram (0.077 ± 0.009 vs. 0.019 ± 0.003, *P* < 0.05; *Figure 2J* and *K*).

### 3.4 Small-molecule inhibition of RUNX1 remedies the HFpEF phenotype

**Figure 3:**
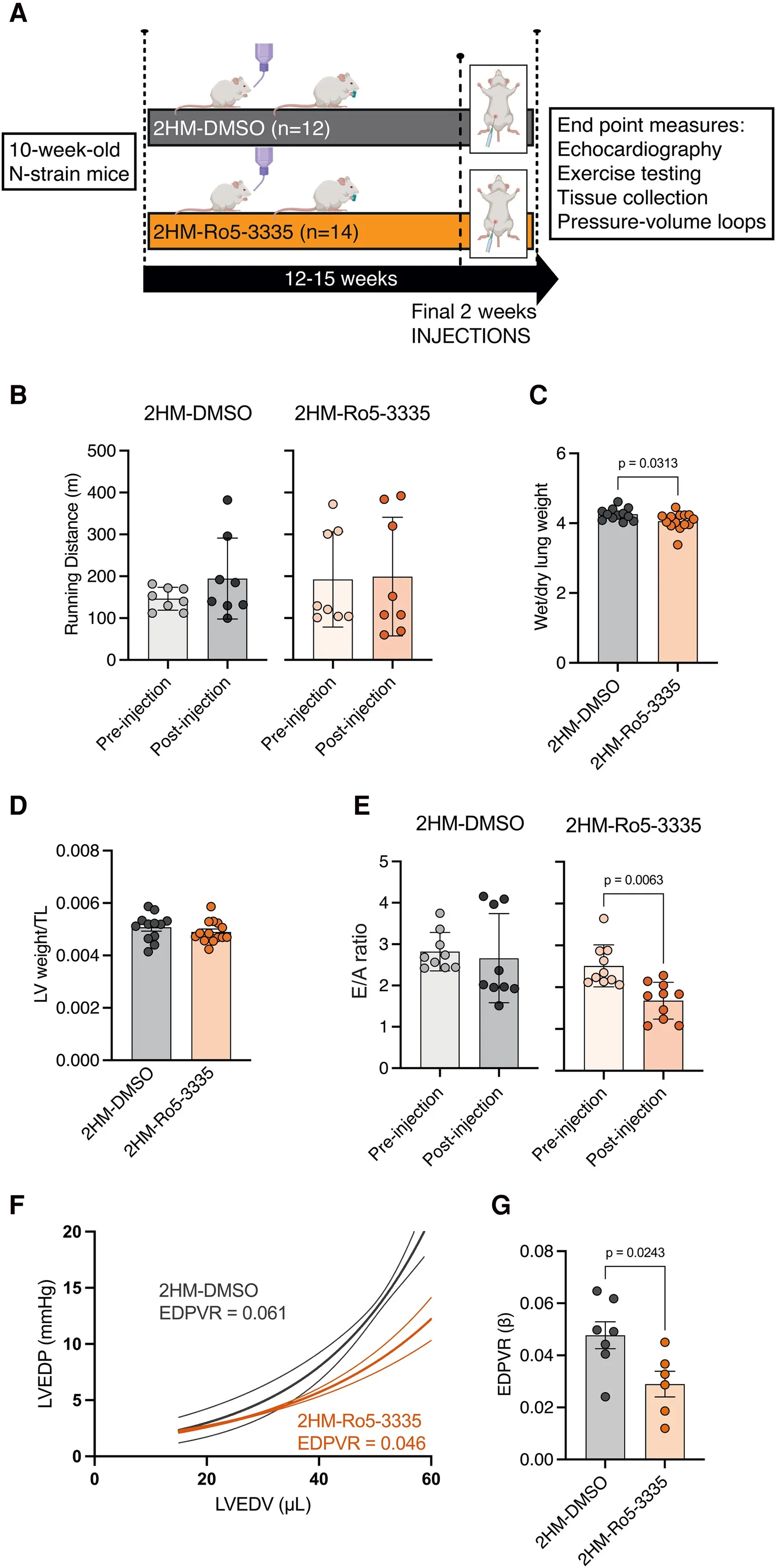
*Runx*1 small-molecule inhibitor Ro5-3335 remedies against diastolic dysfunction. (*A*) Schematic of two-hit protocol and experimental groups, 2HM-DMSO (*n* = 12) and 2HM-Ro5-3335 (*n* = 14), created with BioRender. Characterization of the HFpEF phenotype: (*B*) exercise intolerance was quantified by running distance, pre- and post-treatment (2HM-DMSO: *n* = 8; 2HM-Ro5-3335: *n* = 8) by *t*-test. (*C*) Pulmonary oedema was quantified by wet to dry lung weight ratio (2HM-DMSO: *n* = 12; 2HM-Ro5-3335: *n* = 14) by *t*-test. Hypertrophy was quantified by (*D*) LV weight normalized to TL (2HM-DMSO: *n* = 12; 2HM-Ro5-3335: *n* = 14) by *t*-test. Diastolic function quantified by (*E*) *E*/*A* wave ratio from pulsed wave Doppler echocardiography, pre- and post-treatment (2HM-DMSO: *n* = 9; 2HM- Ro5-3335: *n* = 10) by *t*-test and by the slope (β) of the EDPVR derived from the exponential equation: (LVEDP = curve fitting constant × *e*^[stiffness constant^  ^×^  ^LV end-diastolic volume]^) 2HM-DMSO: *n* = 7; 2-HM-Ro5-3335; *n* = 6 by *t*-test. (*F*) Representative curves (error lines denote 95% confidence interval); and (*G*) data set by *t*-test.

To further translate our findings into a clinically relevant approach, we aimed to identify if inhibition of RUNX1 could reverse the HFpEF phenotype after HFpEF was established using small-molecule inhibitor of RUNX1.^21^ Ten- to 12-week-old C57BL/6N strain male mice were placed on the 2HM protocol for 10–12 weeks. Prior to drug treatment, *in vivo* parameters were utilized to ensure the HFpEF phenotype had developed and any mice that did not have HFpEF symptoms were excluded so that we were only attempting to treat mice with a phenotype to attenuate. Next, while mice remained on 2HM protocol, we injected either a small-molecule inhibitors of RUNX1, Ro5-3335, or vehicle control DMSO every second day during the final two weeks of the protocol prior to collecting end-point measurements and organometrics (*Figure 3A*). Although RUNX1 inhibition by Ro5-3335 injections did not change exercise tolerance (199.1 ± 50.2 vs. 192.5 ± 40.21, *P* > 0.05; *Figure 3B*), pulmonary oedema was reduced in 2HM-Ro5-3335 mice compared with 2HM-DMSO mice (4.26 ± 0.05 vs. 4.06 ± 0.07, *P* < 0.05; *Figure 3C*). As with exercise intolerance, hypertrophy was not changed by Ro5-3335 administration (4.9 × 10^−3^ ± 1.1 × 10^−4^ vs. 5.07 × 10^−3^ ± 1.476 × 10^−4^, *P* > 0.05, *Figure 3D*). Diastolic dysfunction was attenuated in 2HM-Ro5-3335 mice compared with 2HM-DMSO. There was no difference in *E*/*A* wave ratio post-injection compared with pre-injection in the 2HM-DMSO mice (2.66 ± 0.35 vs. 2.82 ± 0.15, *P* > 0.05; *Figure 3E*; Supplementary material online, *Figure S5*) whereas the post-injection E/A wave ratio was reduced in the 2HM-Ro5-3335 mice compared with pre-injection, demonstrating diastolic dysfunction was attenuated (1.68 ± 0.14 vs. 2.51 ± 0.16, *P* < 0.05; *Figure 3E*; Supplementary material online, *Figure S5*). This was confirmed using PV loop assessment of diastolic function by EDPVR in 2HM-DMSO mice compared with the 2HM-Ro5-3335 group (0.048 ± 0.005 vs. 0.029 ± 0.005, *P* < 0.05; *Figure 3F* and *G*).

### 3.5 RNAseq predicts patterns of transcriptional changes consistent with an HFpEF phenotype

**Figure 4:**
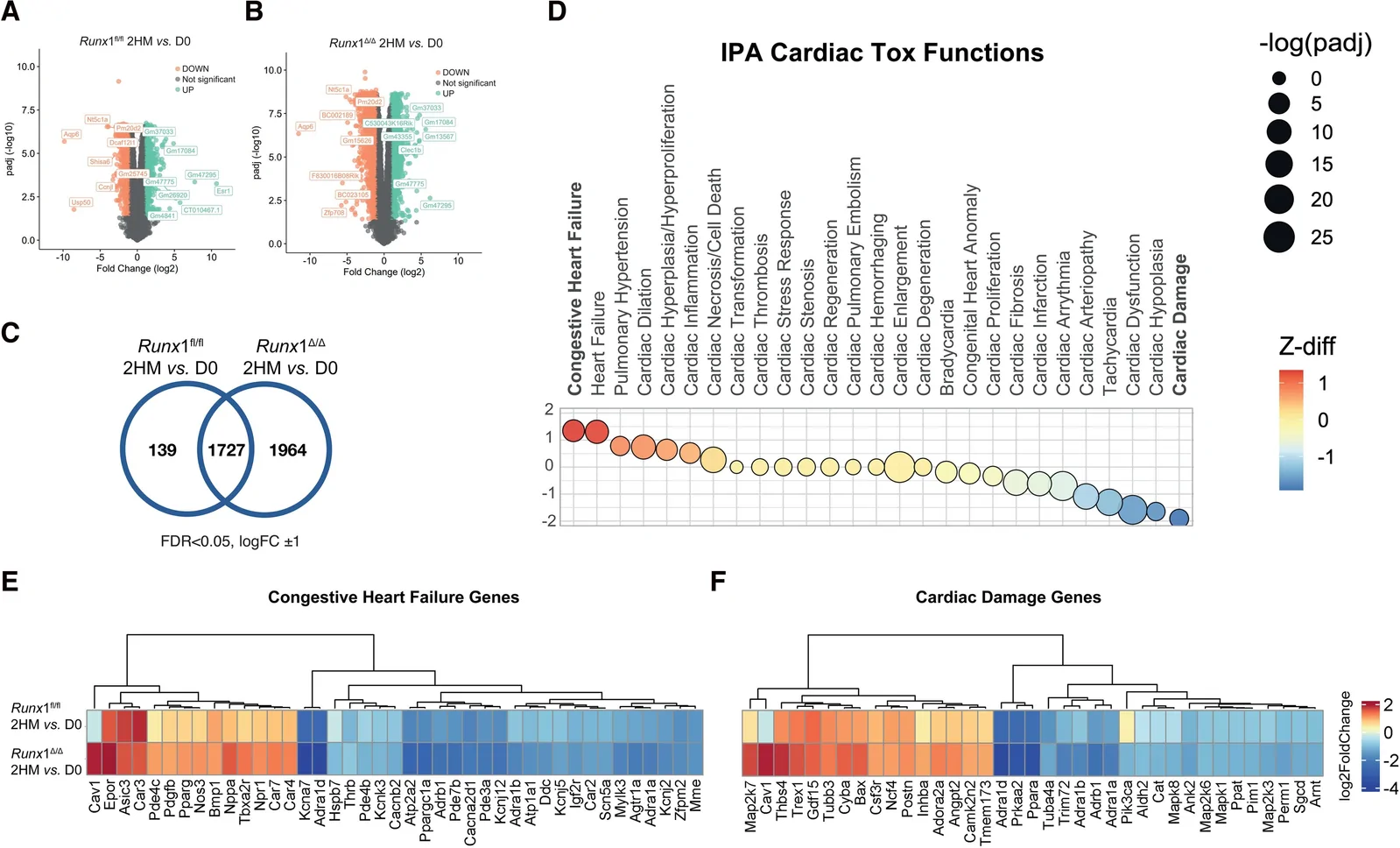
Cardiac DEG analysis of *Runx*1^fl/fl^ and *Runx*1^Δ/Δ^ mice between Day 0 (D0) and 13 weeks of 2HM protocol. Volcano plots of all genes (orange-significantly down-regulated, green-significantly up-regulated and grey not changing) with the eight most regulated genes indicated in (*A*) *Runx*1^fl/fl^ (CTRL: *n* = 6; 2HM: *n* = 6) and (*B*) *Runx*1^Δ/Δ^ mice (CTRL: *n* = 6; 2HM: *n* = 6). (*C*) Venn diagram indicating unique changes and the large number of genes that are commonly differentially regulated between group comparisons. (*D*) Differences in functional predictions using Z-scores comparing *Runx*1^fl/fl^ mice to *Runx*1^Δ/Δ^ mice (red indicating an activation between *Runx*1^fl/fl^ minus *Runx*1^Δ/Δ^, blue indicating an inhibition, and yellow indicating similar functional predictions in both groups). Heat map representing patterns of (*E*) congestive heart failure and (*F*) cardiac damage gene expression levels between *Runx*1^fl/fl^ mice to *Runx*1^Δ/Δ^ mice.

To gain broader insight into the role of *Runx*1 in HFpEF, we performed bulk RNAseq on analysis on LV tissue samples from *Runx1*^fl/fl^ and *Runx1*^Δ/Δ^ mice both at baseline (Day 0, D0) and at the end of the 2HM study. There were not any significantly differentially expressed genes (DEG) between *Runx1*^fl/fl^ and *Runx1*^Δ/Δ^ at D0 and, despite the large phenotypic differences, there were only 32 DEG between *Runx1*^fl/fl^ and *Runx1*^Δ/Δ^ mice at Week 13 (see Supplementary material online, *Table S2*). However, there were many differences when comparing each strain at Week 13 with their respective baseline controls. Thus, because the transcriptomic snapshot at the end of the study does not depict the highly different phenotypes, we focused on comparing the changes from D0 to the end time point within each strain. Using a false discovery rate cut-off of ≤0.05 and log-fold change (logFC) ± 1, there were 1866 DEG in 2HM-*Runx1*^fl/fl^ mice at Week 13 compared with D0 *Runx1*^fl/fl^ mice (*Figure 4A* and *C*) and 3691 DEG at Week 13 in 2HM-*Runx*1^Δ/Δ^ mice compared with the D0 (*Figure 4B* and *C*). The majority of DEG were shared between strains (1727 DEG: 92.6% of total DEG for *Runx1*^fl/fl^ and 53.2% of total DEG for *Runx*1^Δ/Δ^; *Figure 4C*). Interestingly, the unique changes in the *Runx*1^Δ/Δ^ mice across time points may account for the large functional differences observed because there were very few unique changes in the *Runx1*^fl/fl^ mice (*Figure 4C*). Using all significantly DEG in *Runx1*^fl/fl^ and *Runx1*^Δ/Δ^ mice at Week 13 compared with baseline, we focused on cardiac toxicity functions defined by IPA software. We compared *Z*-scores (a statistical measure utilized to determine the significance and directionality of gene expression changes within a given pathway over that time course) from *Runx1*^fl/fl^ (Week 13 of 2HM vs. D0) and *Runx1*^Δ/Δ^ (Week 13 of 2HM vs. D0). We visualized the impact of *Runx*1 deficiency by plotting the difference between *Z*-scores from *Runx1*^fl/fl^ (2HM vs. D0) minus Runx1^Δ/Δ^ (2HM vs. D0) mice (*Z*-diff; *Figure 4D*). While some predictive changes in cardiac toxicity functions demonstrated limited difference in *Z*-diff (yellow; *Figure 4D*), the largest differences in *Z*-score were in congestive heart failure genes and cardiac damage genes. The genes included by IPA in these two cardiac toxicity functions were then plotted using a heat map (*Figure 4E* and *F*).

### 3.6 Inhibition of *Runx*1 in female mice: reversal of HFpEF phenotype

**Figure 5:**
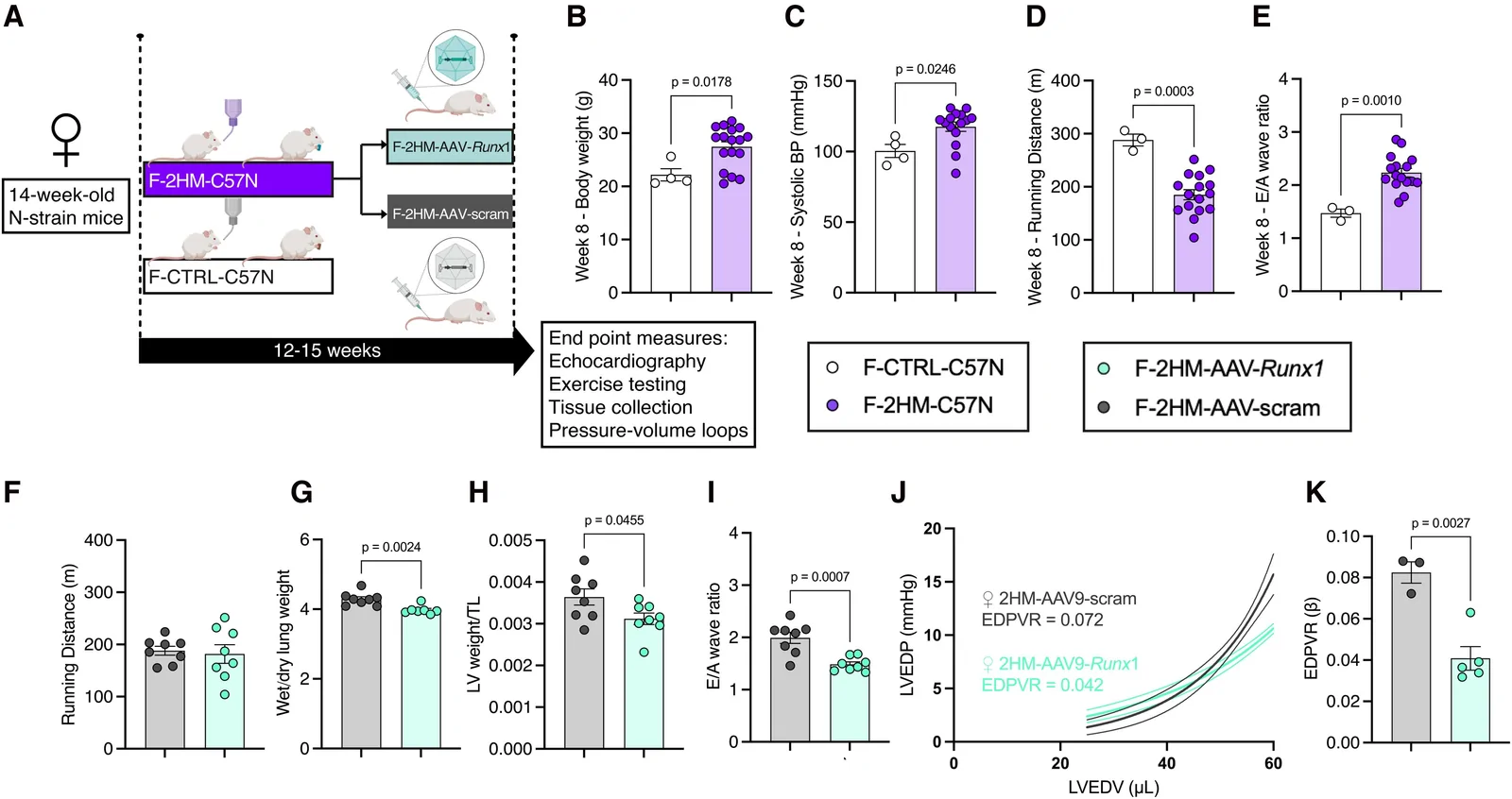
AAV9-mediated knockdown of *Runx*1 in female mice: partial reversal of HFpEF phenotype. (*A*) Schematic of two-hit protocol and experimental groups, F-CTRL-C57N (*n* = 4), F-2HM-C57N (*n* = 16: F2HM-AAV-scram: *n* = 8; F2HM-AAV-*Runx*1: *n* = 8), created with BioRender. (*B*) Body weight (F-CTRL-C57N: *n* = 4; F-2HM-C57N, *n* = 17) by *t*-test. (*C*) SBP (F-CTRL-C57N: *n* = 4; F-2HM-C57N: *n* = 15) by *t*-test. (*D*) Exercise intolerance testing quantified by running distance (F-CTRL-C57N: *n* = 3; F-2HM-C57N: *n* = 16) by *t*-test. Diastolic function quantified by (*E*) *E*/*A* wave ratio from pulsed wave Doppler echocardiography at Week 8 (F-CTRL-C57N: *n* = 3; F-2HM-C57N: *n* = 16) by *t*-test. Post-injection, (*F*) exercise intolerance was quantified by running distance (F-2HM-scram: *n* = 8; F-2HM*-Runx*1: *n* = 8) by *t*-test. (*G*) Pulmonary oedema quantified by wet to dry lung weight ratio following removal of outlier identified by ROUT outlier test (F-2HM-scram: *n* = 8; F-2HM*-Runx*1: *n* = 7). Hypertrophy was quantified by (*H*) LV weight normalized to TL (F-2HM-scram: *n* = 8; F-2HM*-Runx*1: *n* = 8) by *t*-test. Diastolic function quantified by (*I*) *E*/*A* wave ratio from pulsed wave Doppler echocardiography (F-2HM-scram: *n* = 8; F-2HM*-Runx*1: *n* = 8) and by the slope (β) of the EDPVR derived from the exponential equation: LVEDP = curve fitting constant × *e*^[stiffness constant^  ^×^  ^LV end-diastolic volume]^ (F-2HM-scram: *n* = 3; F-2HM*-Runx*1: *n* = 5) by *t*-test. (*J*) Representative curves (error lines denote 95% confidence interval); and (*K*) data set by *t*-test.

To further increase the relevance and translational impact of our findings, we expanded our study in two ways: we used female mice to ensure clinical relevance; and we waited to intervene with *Runx*1 inhibition *via* RNA interference until HFpEF was already established in the mice, to test its utility to reverse adverse cardiac remodelling in HFpEF. It has been demonstrated that it is more difficult to induce an HFpEF phenotype *via* the 2HM in female mice compared with males in young mice.^22^ Thus, in a cohort of C57N strain females we waited until they were aged 14 weeks (∼40% older than previous data) before placing them on the 2HM protocol with a ramping dose of L-NAME (*Figure 5A*). Once again, we ensured efficacy of the two-hits by measuring body weight and SBP in a female CTRL-C57N group (F-CTRL-C57N, *n* = 4) compared with a female 2HM-C57N group (F-2HM-C57N, *n* = 16; *Figure 5B* and *C*). We utilized our intermediary *in vivo* phenotypic measures exercise intolerance (*Figure 5D*) and diastolic dysfunction (*Figure 5E*) to confirm that at the 8-week time point the F-2HM-C57N group had established an HFpEF phenotype. Following this, we split the F-2HM-C57N group into two groups for AAV-mediated gene delivery such that they had consistent starting parameters (*Figure 5A*). One group was injected with AAV9-scramble-shRNA (F-2HM-AAV9-scram, *n* = 8) and a second injected with AAV9-*Runx*1-shRNA to knockdown *Runx*1 (F-2HM-AAV9-*Runx*1, *n* = 8). Consistent with the male AAV study, there were no differences in running distance between groups 4 weeks following AAV injection (F-2HM-AAV9-scram: 188 ± 8 m, F-2HM-AAV9-*Runx*1: 182 ± 17 m, *P* = 0.7560; *Figure 5F*). We found pulmonary oedema was reduced in the F-2HM-AAV9-*Runx*1 compared with F-2HM-AAV9-scram (3.97 ± 0.05 vs. 4.29 ± 0.07, respectively, *P* = 0.0024; *Figure 5G*) which was consistent with the male data (*Figure 2E*). Although hypertrophy (measured by LV weight normalized to TL) was not different between the 2HM-AAV-scram and 2HM-AAV-*Runx*1 males (*Figure 2F*), it was reduced in F-2HM-AAV9-*Runx*1 compared with F-2HM-AAV-scram (3.1 ± 0.1 × 10^−3^ vs. 3.6 ± 0.2 × 10^−3^, respectively, *P* < 0.05; *Figure 5H*). Finally, diastolic dysfunction was attenuated as measured both by E/A wave ratio from pulse wave Doppler echocardiography (F-2HM-AAV-scram: 1.99 ± 0.11 vs. F-2HM-AAV9-*Runx*1: 1.49 ± 0.05, *P* = 0.0007 *Figure 5I*; Supplementary material online, *Figure S6*), and by the slope of the EDPVR (F-2HM-AAV-scram: 0.082 ± 0.0002 vs. F-2HM-AAV9-*Runx*1: 0.041 ± 0.0057, *P* = 0.0027; *Figure 5J* and *K*).

## 4. Discussion

This work identifies a critical role for RUNX1 in the development of HFpEF. Furthermore, we provide evidence that targeting *Runx*1 in the context of HFpEF has clinical translational potential.

In recent years, significant work has been done to establish a model with preserved EF which not only demonstrates increased hypertrophy but also phenotypes such as pulmonary oedema, exercise intolerance, and diastolic dysfunction and therefore is more representative of the multi-morbidity, multi-system disorder of HFpEF in humans.^19,23^

*Runx*1 has been robustly demonstrated to play an important role in the context of cardiac disease, with a particular emphasis on its importance in adverse cardiac remodelling following MI.^10,21,24,25^ Previous work has demonstrated the beneficial effects of targeting *Runx*1 in the context of acute MI^21^ and in the context of ischaemic heart disease; however, whether these benefits would be observed in a cardiac syndrome of a chronic progressive nature such as HFpEF was unknown.

Therefore, we adapted a 2HM of HFpEF in our line of transgenic mice with cardiomyocyte *Runx*1 deficiency, and then again with the C57-*N* strain mice using translational approaches to target *Runx*1. Overall, this work has identified RUNX1 as a promising new therapeutic target for treatment and prevention of HFpEF.

Targeting *Runx*1 with a cardiomyocyte-specific *Runx*1-deficient mouse attenuates the development of an HFpEF phenotype. *Runx*1-deficiency is highly protective against the development of HFpEF because despite the efficacy of the two-hits (i.e. mice in both 2HM groups gained weight and had increased SBP), the *Runx*1-deficient mice did not develop all the signs of HFpEF whereas control mice had a classical HFpEF phenotype. Specifically, *Runx*1 deficiency reduced the development of hypertrophy and exercise intolerance and completely protected against development of pulmonary oedema and diastolic dysfunction. Although in this study we have simply targeted a single gene (*Runx*1) in a single-cell type (cardiomyocytes), the phenotypic outcome was evident systemically including effects on exercise intolerance, pulmonary oedema, and the mass of peripheral organs, reflecting the beneficial effects of targeting *Runx*1 for both cardiac dysfunction and peripheral systems.

We corroborated these findings using RNAi. Interestingly, similar to the convincing protection of *Runx*1-deficient mice, targeting *Runx*1 with RNAi also protected mice against the development of diastolic dysfunction and pulmonary oedema. While these results are promising, we utilized a preventive strategy, which is not necessarily applicable to all clinical scenarios. An interventional approach was therefore used by administering a small-molecule RUNX1 inhibitor Ro5-3335, *after* the establishment of HFpEF. This approach to inhibit Runx1 demonstrated similar outcomes, albeit with fewer parameters affected. This difference in outcomes may reflect the number of cardiomyocytes exposed to the intervention and/or, effects on non-cardiomyocytes or duration of exposure. For example, the difference in cardiomyocyte cross-sectional area despite the lack of change in LV mass in 2HM-AAV9-*Runx*1 compared with 2HM-AAV9-scram may be due to technical variability in LV isolation or may point to mechanistic differences, such as increased extracellular matrix or interstitial content, or altered cellular composition. Future work will aim to further understand the relative benefits of different approaches.

To interrogate potential gene changes underlying the phenotypic differences observed when inhibiting *Runx*1, we used RNAseq. This resulted in predictions using IPA software for changes in the regulation of diseases and functions when comparing the final time point (after 13 weeks of 2HM) tissue in both groups compared with Day 0 (D0) heart tissues. In any chronic disease it is difficult to determine at which time point transcriptional changes might best be identified in order to discern differences between *Runx1*^fl/fl^ and *Runx1*^Δ/Δ^ mice because relevant changes in the transcriptome may precede phenotype differences. As such, it is perhaps unsurprising that the most DEG were shared between strains despite the stark phenotypic differences between the 2HM transgenic groups. However, IPA did predict an upregulation in congestive heart failure pathways in both *Runx1*^fl/fl^ and *Runx1*^Δ/Δ^ mice, with larger changes occurring in *Runx1*^fl/fl^ compared with *Runx1*^Δ/Δ^ mice despite more gene changes overall occurring in the *Runx1*^Δ/Δ^ mice. Interestingly, it was predicted that the upregulation of cardiac damage pathways would result in larger changes in *Runx1*^Δ/Δ^ mice compared with *Runx1*^fl/fl^ mice.

Finally, we expanded the translational relevance of our work by performing a study to reverse adverse cardiac remodelling in HFpEF in female mice. This enabled us to not only determine the effect of gene transfer in both sexes but also interrogate the translational potential of targeting *Runx*1 with AAV9 after the HFpEF phenotype was fully established (in contrast to our male study where AAV was administered prior to mice being placed on the 2HM protocol). Overall, inhibiting *Runx*1 *via* RNAi was effective in reducing hypertrophy, pulmonary oedema, and reversing diastolic dysfunction in the female 2HM, thus indicating a potential role for *Runx*1 in the treatment of HFpEF in both females as well as males.

The aetiology of HFpEF and the associated changes in heart structure and diastolic function are complex and relatively poorly understood. The relative contributions of the metabolic changes at a cellular level and the chronic low-grade inflammation that accompanies metabolic stress and hypertension are not clear. It is remarkable that the relatively simple model developed by Schiattarella *et al.* in 2019 and used again here can recapitulate many of the phenotypic changes associated with HFpEF given the subtleties of these physiological insults and their complex interplay. We acknowledge that RUNX1 is likely to modulate several aspects in the heart that mediate the pathogenesis of this syndrome. Notably, the cardioprotection mediated by *Runx1-*deficiency was independent of changes in blood pressure or body weight, demonstrating that its beneficial effects are not mediated through the attenuation of primary syndrome inducers but rather through direct modulation of the cardiac response to such stress. It is intriguing that attenuation of *Runx*1 function alleviates the deleterious effects observed both following MI and prevents and reverses key aspects of HFpEF, hinting at a more fundamental role in the response of heart damage and pathophysiological insult. It is plausible that RUNX1 acts as a maladaptive master regulator across heart failure, with context-specific actions. In HFrEF, often driven by acute cardiac injury, RUNX1 compromises systolic function.^5–7^ In HFpEF, which is characterized by chronic metabolic and hypertensive stress, it promotes diastolic dysfunction. This phenotypic divergence, may arise from pathology-specific upstream signals engaging distinct transcriptional programmes, as supported by our RNAseq data. However, we note that there are common pathways that are involved in both HFrEF and HFpEF (abnormal calcium handling, mitochondrial function, and inflammation/immune response) that Runx1 can target in both syndromes and is the focus of our future work.

Overall, this study clearly demonstrates that RUNX1 drives pathological changes in cardiomyocytes in the context of HFpEF. Inhibition of *Runx*1 by gene transfer or the use of a small-molecule inhibitor improves LV diastolic function and represents an exciting translational approach for the treatment of HFpEF.

## 5. Limitations

To assess the role of Runx1 in HFpEF, we induced HFpEF by increasing body weight and blood pressure *via* administration of HFD and L-NAME. This limits our capacity to interrogate the role of Runx1 in the development of hypertension and obesity. In order to gain a more complete understanding of the possible therapeutic potential of Runx1, further work will need to be performed to understand its contribution to this complex and systemic syndrome, including the comorbidities associated with it, such as diabetes and hypertension.

It is possible that many of the DEG in our bulk tissue samples will be the result of transcriptional changes in non-cardiomyocyte cell types in the ventricle, potentially diluting cardiomyocyte-specific changes that are the result of the *Runx*1 deficiency. Single-cell transcriptomic analysis, potentially at multiple time points, is part of the programme of future work.

Additionally, AAV9-mediated *Runx1* knockdown approach in male mice was prophylactic, which does not mirror a typical clinical scenario albeit the female study clearly demonstrates that inhibition of Runx1 can reverse diastolic dysfunction in HFpEF. Furthermore, the small-molecule inhibitor Ro5-3335, while administered after onset of the HFpEF phenotype, showed a more restricted improvement, primarily reversing diastolic dysfunction and reducing pulmonary oedema. We note also that echocardiographic assessment of diastolic function was performed without simultaneous ECG recording and exercise capacity was not normalized to body weight and should be taken into consideration in future studies exploring the benefits of Runx1 inhibitors in cardiac disease.

Translational perspective HF is a leading cause of death worldwide and traditionally divided into different subtypes according to cardiac EF. In contrast to HFrEF, there are limited treatment options for HFpEF which is of considerable concern given that HFpEF is projected to become the dominant HF subtype in the future.^26^ RUNX1 has been demonstrated to play an important role in the development of many cardiac and non-cardiac diseases. As a result, the potential for RUNX1 inhibitors as therapeutic agents across various conditions has become increasingly evident. In this study, we established the therapeutic potential of targeting RUNX1 in the context of HFpEF. Targeting RUNX1 using different strategies markedly attenuates the development of diastolic dysfunction and pulmonary oedema in HFpEF. Therefore, RUNX1 represents a novel translational therapeutic target with great potential to address one of the biggest challenges in cardiac research.

## Supplementary Material

cvag106_Supplementary_Data

## Data Availability

Data from this study are available upon request to the corresponding author.
