# Supplementary material for: Targeting RUNX1 protects against diastolic dysfunction in a two-hit mouse model of heart failure with preserved ejection fraction
Source: Cardiovasc Res. 2026 May 26;122(10):1318–28. doi: 10.1093/cvr/cvag106 (PMC13355837; doi:10.1093/cvr/cvag106)
Supplement: cvag106_Supplementary_Data [file cvag106_supplementary_data.zip › SUPPLEMENTAL_26JAN2026.pdf]

SUPPLEMENTAL FIGURE 1

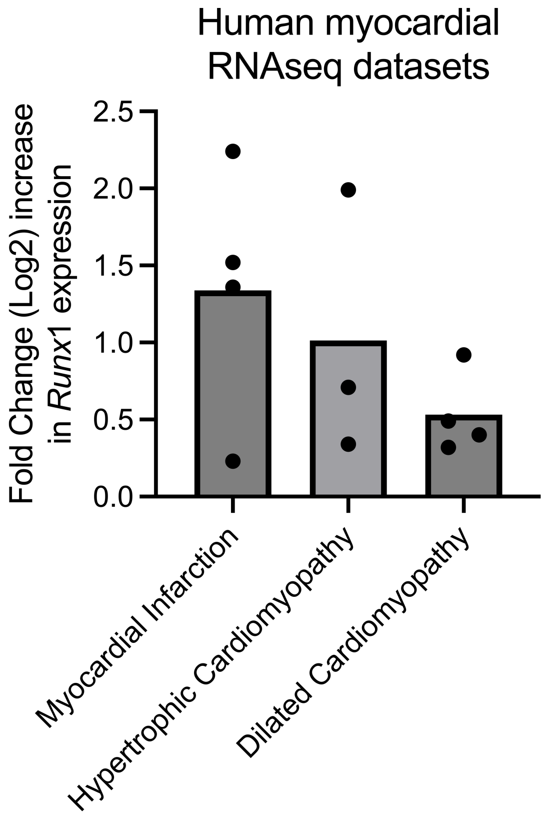

# SUPPLEMENTAL FIGURE 2

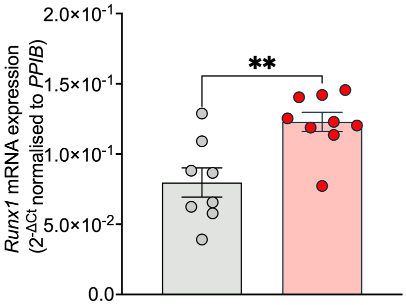

SUPPLEMENTAL FIGURE 3

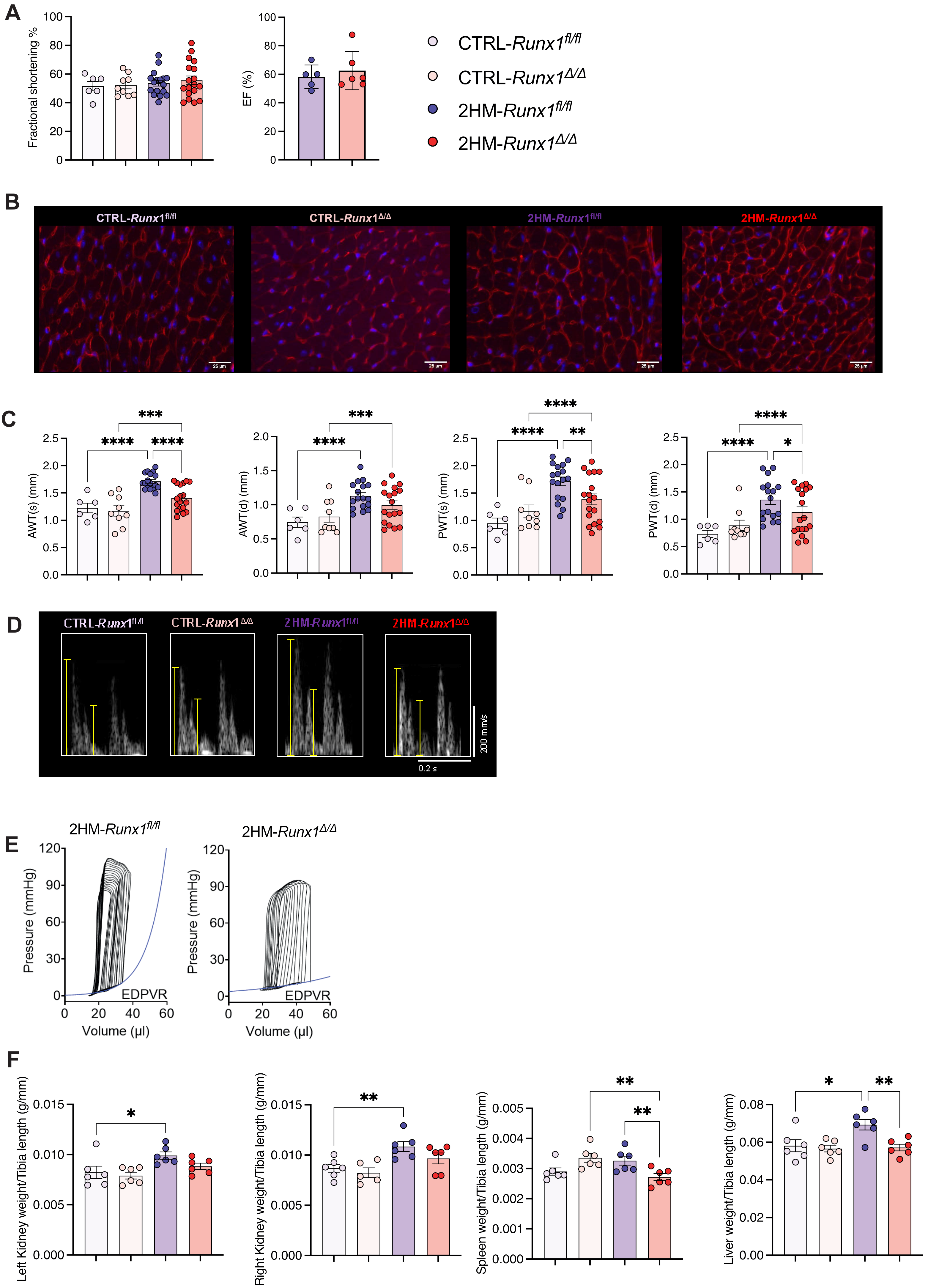

SUPPLEMENTAL FIGURE 4

A

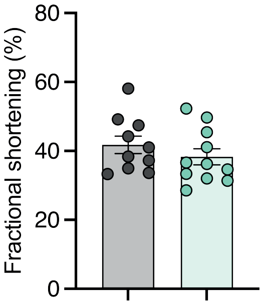


B

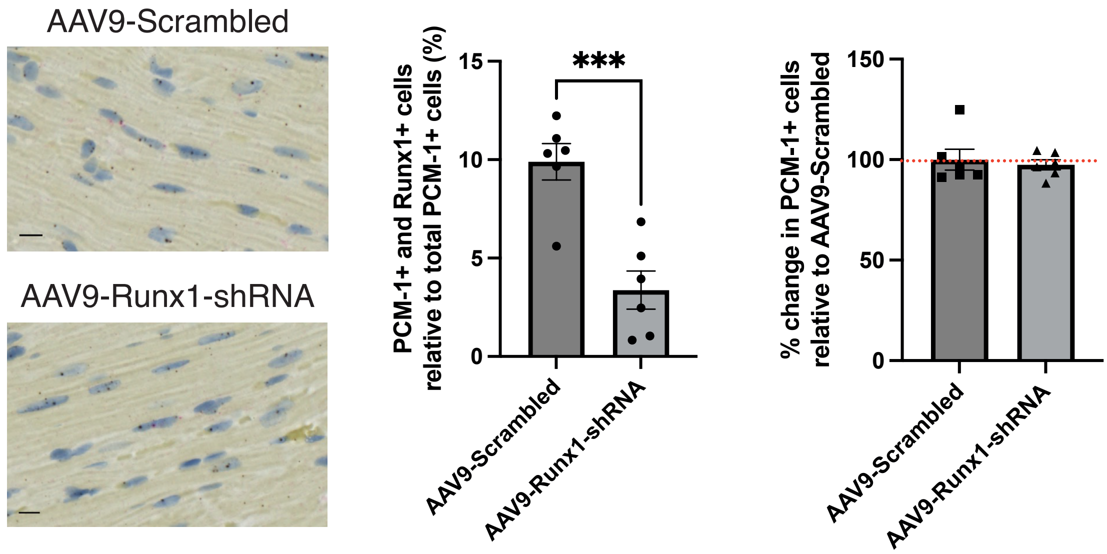


C

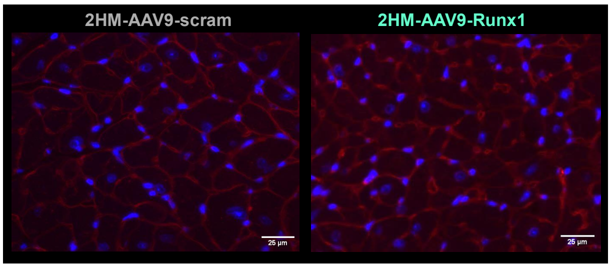


D

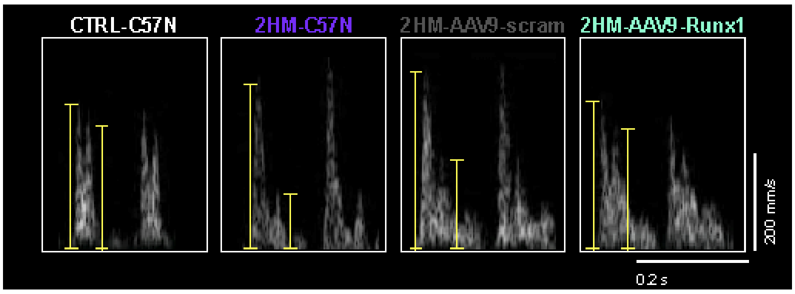

SUPPLEMENTAL FIGURE 5

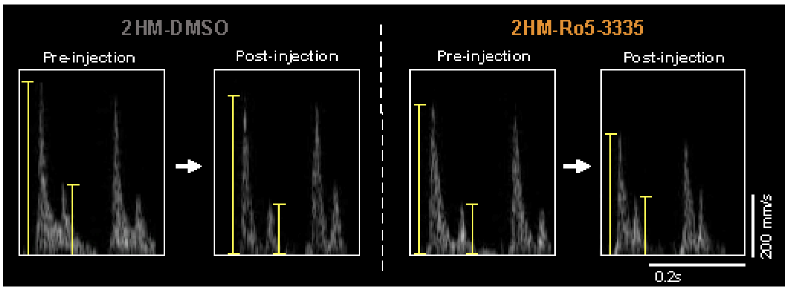

# SUPPLEMENTAL FIGURE 6

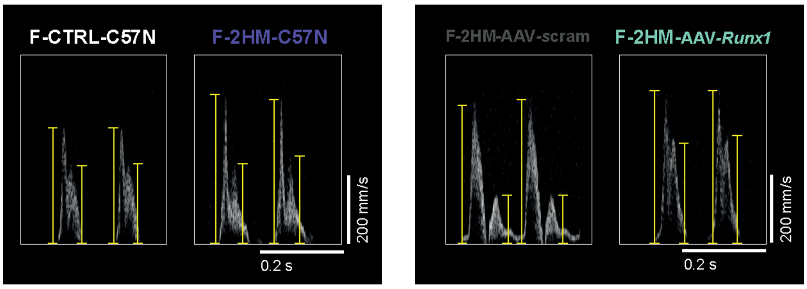

SUPPLEMENTAL TABLE 1

| ComparisonID | GeneName | ComparisonContrast | Pvalue | Adjusted Pvalue | Fold Change [Log2] | Fold change (UP) | Case.DiseaseState |
|---|---|---|---|---|---|---|---|
| Myocardial Infarction | | | | | | | |
| GSE120852.GPL16791.DESeq2R.test3 | RUNX1 | ExperimentGroup => right ventricular tissue from ICM, biventricular heart failure vs right v | 0.0005 | 0.0097 | 2.24 | 4.74 | heart failure;myocardial ischemia |
| GSE116250.GPL16791.DESeq2R.test7 | RUNX1 | Gender:DiseaseState => male -> myocardial ischemia vs disease control | 0.0019 | 0.011 | 1.52 | 2.88 | myocardial ischemia |
| GSE116250.GPL16791.DESeq2R.test2 | RUNX1 | DiseaseState => myocardial ischemia vs disease control | 0.0014 | 0.0072 | 1.36 | 2.57 | myocardial ischemia |
| GSE1145.GPL570.test4 | RUNX1 | DiseaseState => myocardial ischemia vs normal control | 0.0009 | 0.0063 | 0.23 | 1.18 | myocardial ischemia |
| Hypertrophic Cardiomyopathy | | | | | | | |
| GSE89714.GPL11154.DESeq2R.test1 | RUNX1 | DiseaseState => hypertrophic cardiomyopathy vs normal control | 1.41E-08 | 8.01E-07 | 1.99 | 3.98 | hypertrophic cardiomyopathy |
| GSE141910.GPL16791.DESeq2R.test2 | RUNX1 | DiseaseGroup => hypertrophic cardiomyopathy vs non-failing donor | 0.0006 | 0.0033 | 0.71 | 1.64 | hypertrophic cardiomyopathy |
| GSE1145.GPL570.test2 | RUNX1 | DiseaseState => hypertrophic cardiomyopathy vs normal control | 0.0016 | 0.0152 | 0.34 | 1.27 | hypertrophic cardiomyopathy |
| Dilated Cardiomyopathy | | | | | | | |
| GSE35108.GPL6244.test4 | RUNX1 | Transfection:DiseaseState => GFP -> dilated cardiomyopathy vs normal control | 0.0002 | 0.0021 | 0.92 | 1.89 | dilated cardiomyopathy |
| GSE35108.GPL6244.test3 | RUNX1 | Transfection:DiseaseState => ATP2A2 -> dilated cardiomyopathy vs normal control | 0.0022 | 0.0095 | 0.49 | 1.41 | dilated cardiomyopathy |
| GSE141910.GPL16791.DESeq2R.test1 | RUNX1 | DiseaseGroup => dilated cardiomyopathy;heart failure vs non-failing donor | 0.0004 | 0.0015 | 0.40 | 1.32 | dilated cardiomyopathy;heart failure |
| GSE155495.GPL21697.DESeq2R.test1 | RUNX1 | DiseaseState => dilated cardiomyopathy;heart failure vs disease control | 0.0121 | 0.0436 | 0.32 | 1.25 | dilated cardiomyopathy;heart failure |

SUPPLEMENTAL TABLE 2

© 2000-2024 QIAGEN. All rights reserved.

| Expr False D | Expr Log | Expr Inte | ID | Symbol | Entrez Gene | Location | Type(s) |
|---|---|---|---|---|---|---|---|
| 0.000021 | -1.709 | 4.215 | ENSMUSG00000036103 | COLEC12 | collectin subfa | Plasma Memb | transmembrane receptor |
| 0.000798 | -1.956 | 5.259 | ENSMUSG00000039852 | RERE | arginine-gluta | Nucleus | transcription regulator |
| 0.00168 | -0.962 | 4.511 | ENSMUSG00000020412 | ASCC2 | activating sigr | Nucleus | other |
| 0.00237 | -1.554 | 4.988 | ENSMUSG00000028995 | HYCC1 | hyccin PI4KA | Cytoplasm | other |
| 0.00237 | 0.914 | 6.84 | ENSMUSG00000058135 | GSTM5 | glutathione S | Cytoplasm | enzyme |
| 0.00791 | 1.174 | 5.201 | ENSMUSG00000039943 | PLCB4 | phospholipas | Cytoplasm | enzyme |
| 0.0121 | -1.289 | -0.253 | ENSMUSG00000086233 | Gm11816 | | Other | other |
| 0.0141 | -0.416 | 5.796 | ENSMUSG00000025369 | SMARCC2 | SWI/SNF rela | Nucleus | transcription regulator |
| 0.0141 | -0.817 | 1.858 | ENSMUSG00000041961 | ZNRF3 | zinc and ring | Plasma Memb | enzyme |
| 0.0259 | 0.744 | 3.852 | ENSMUSG00000027692 | TNIK | TRAF2 and N | Plasma Memb | kinase |
| 0.0259 | 0.788 | 2.593 | ENSMUSG00000024563 | SMAD2 | SMAD family | Nucleus | transcription regulator |
| 0.0266 | -2.594 | 1.017 | ENSMUSG00000098022 | ZFP82 | ZFP82 zinc fi | Nucleus | transcription regulator |
| 0.0274 | -1.197 | 7.892 | ENSMUSG00000025809 | ITGB1 | integrin subur | Plasma Memb | transmembrane receptor |
| 0.0303 | -2.182 | 2.224 | ENSMUSG00000029863 | CASP2 | caspase 2 | Cytoplasm | peptidase |
| 0.0303 | -0.743 | 1.824 | ENSMUSG00000040747 | CD53 | CD53 molecu | Plasma Memb | other |
| 0.0303 | 1.474 | 3.733 | ENSMUSG00000030532 | HDDC3 | HD domain cc | Other | other |
| 0.0303 | 1.285 | 4.582 | ENSMUSG00000022257 | LAPTM4B | lysosomal pro | Cytoplasm | other |
| 0.0303 | -1.197 | 5.872 | ENSMUSG00000035545 | LENG8 | leukocyte rec | Other | other |
| 0.0303 | 1.123 | 4.33 | ENSMUSG00000021518 | PTDSS1 | phosphatidyls | Cytoplasm | enzyme |
| 0.0303 | 1.512 | 6.343 | ENSMUSG00000039844 | RAPGEF1 | Rap guanine | Cytoplasm | other |
| 0.0303 | -0.866 | 1.482 | ENSMUSG00000103651 | Gm37206 | | Other | other |
| 0.0303 | -0.914 | 4.396 | ENSMUSG00000053931 | CNN3 | calponin 3 | Cytoplasm | other |
| 0.0303 | -0.602 | 2.74 | ENSMUSG00000031239 | ITM2A | integral memb | Plasma Memb | other |
| 0.0303 | 0.464 | 4.206 | ENSMUSG00000026047 | POGLUT2 | protein O-gluc | Cytoplasm | enzyme |
| 0.0303 | 1.616 | 5.365 | ENSMUSG00000030861 | ACADSB | acyl-CoA deh | Cytoplasm | enzyme |
| 0.031 | -1.155 | 4.395 | ENSMUSG00000032724 | ABTB2 | ankyrin repea | Nucleus | other |
| 0.031 | -1.41 | 3.626 | ENSMUSG00000059439 | BCAS3 | BCAS3 microt | Nucleus | other |
| 0.031 | -1.329 | 2.405 | ENSMUSG00000008090 | FGFRL1 | fibroblast grov | Plasma Memb | transmembrane receptor |
| 0.031 | 1.648 | 3.078 | ENSMUSG00000037692 | AHDC1 | AT-hook DNA | Nucleus | transcription regulator |
| 0.031 | 0.843 | 4.987 | ENSMUSG00000029647 | PAN3 | poly(A) specif | Cytoplasm | enzyme |
| 0.0362 | -0.417 | 3.396 | ENSMUSG00000081272 | Ap2m1-ps | adaptor-relate | Other | other |
| 0.0391 | -1.994 | 1.382 | ENSMUSG00000106296 | 4632404M16 | RIKEN cDNA | Other | other |
| 0.0391 | -0.944 | 5.655 | ENSMUSG00000053907 | MAT2A | methionine ac | Cytoplasm | enzyme |
