# Supplementary material for: Targeting RUNX1 protects against diastolic dysfunction in a two-hit mouse model of heart failure with preserved ejection fraction
Source: Cardiovasc Res. 2026 May 26;122(10):1318–28. doi: 10.1093/cvr/cvag106 (PMC13355837; doi:10.1093/cvr/cvag106)
Supplement: cvag106_Supplementary_Data [file cvag106_supplementary_data.zip › SUP_METHODS_30APRIL26.docx]

**SUPPLEMENTAL METHODS**

**Animals**

The care and use of animals were in accordance with the UK Government Animals (Scientific Procedures) Act 1986. All animal procedures were approved by the University of Glasgow Animal Welfare and Ethical Review Body and licensed by the Home Office, UK (project licence no. P05FEIF82 and subsequently PP4465428). Mice were housed on a 12/12 h light/dark cycle and fed and watered *ad libitum*. Animals used were male and female mice aged 10-15 weeks of age (weight, 25-30 g) and were assigned to experimental groups such that baseline body weight was not different between groups. Animals were humanly killed under inhalational anaesthesia by schedule one procedures (cervical dislocation).

**Data and Statistics**

Data are either expressed as mean ± SEM or in the dot plot and bar graph combined graphs as individual mice (dots) and averages (bar graphs) ± SEM. Comparisons between two experimental groups were performed with the Student’s t-test on raw data. Comparisons between more than two groups were conducted on raw data with ANOVA (Graph Pad Prism 9 software).

**Generation of 2-hit HFpEF mouse model**

We generated a 2-hit mouse model (2HM) of HFpEF as previously described^1,2^. Mice were given 60% Kcal/100 g high-fat diet (HFD, Research Diets Inc, USA) to induce weight gain and metabolic disturbance and L-NAME (N_ω_-Nitro-L-arginine methyl ester hydrochloride, Merck Life Sciences UK Limited) in drinking water in opaque water bottles (Tecniplast, UK) to increase blood pressure. The HFD was changed every 2 weeks and L-NAME was changed every 2 days. Control mice were fed a standard chow diet (SDS diets, UK) and tap water.

**Blood pressure training and measurement**

BP-2000 Blood Pressure Analysis System™ (BiosebLab instruments, France) was utilised for tail-cuff mice blood pressure measurements. Animals were handled calmly during the procedure to minimize stress. Animals underwent 2-3 training sessions separated by 24 hours that involved acclimatization in the heating chamber, restraint and application of the tail cuff. . The actual blood pressure measurement was taken the day after the last training session. For this, animals became acclimatized to the chamber temperature and light for 10 min after which blood pressure measurements were recorded for each mouse. An average of 10-20 readings was taken as a representative for the time point measurement every week for each animal.

**Echocardiography**

Mice were anesthetized using a pre-filled induction chamber with 5% isoflurane (Isoflo, Abbott Laboratories, USA) in 1.0 L/min O_2_ and subsequently moved to a face mask, where anaesthesia was maintained at 0.5-1% isoflurane in 1.0 L/min O_2_. Echocardiography was carried out using either a Siemens ACUSON Seqoula C512 and a 15L8 transducer or a Visual Sonics VEVO F2 with a UHF57x transducer. Left ventricular (LV) internal diameter (LVID), anterior wall thickness (AWT) and posterior wall thicknesses (PWT) at both diastole (d) and systole (s) were measured from transverse M-mode echocardiography images at the papillary muscle level. LV fractional shortening (FS) was calculated using the following formula:

$$FS \left( \% \right)=\frac{(LVIDd-LVIDs)}{LVIDd} x 100$$

E wave velocity (peak doppler blood inflow velocity across the mitral valve during early diastole) and A wave velocity (peak doppler blood inflow velocity across the mitral valve during late diastole) were acquired from pulsed-wave (PW) doppler mode. E and A waves velocities were calculated by measuring the height of each wave from base to peak and the E/A ratio was then determined. An average of at least three waves per mouse was taken.

**Exercise intolerance assessment**

Forced exercise walking wheel (model 80800A; Lafayette instruments, UK) was utilised to evaluate exercise intolerance in animals. Mice were acclimatised to the exercise wheel for three consecutive days before the intolerance test. On the first day, mice were placed inside the stationary exercise wheel for 10 min. On the second day, mice were trained to walk 3 m/min for 5 min. On the third day, mice were trained to run 4 m/min for 10 min. For the intolerance test, animals started at a 3 m/min warm-up speed for 5 min after which the speed was increased to 4 m/min until exercise intolerance. Exercise intolerance was defined as the inability of the animal to resume running within 10 seconds of coasting inside the wheel.

**Pressure Volume (PV) Loop measurements**

We used 1.2F 3.5 mm PV catheter (ADV500, Transonic) to perform the experiments. It is an invasive tool for real-time assessment of cardiac function through visualisation of LV pressure plotted against LV volume. Mice were anaesthetised with 5% isoflurane in the induction chamber and then maintained on a face mask (1.5-2% isofluorane). The skin from the neck area was shaved and cleaned with chlorohexidine (Hibiscrub, Ecolab Ltd, UK). Mice were placed supine on a thermostatically controlled heat pad with a rectal probe to monitor body temperature at 37°C (Harvard Apparatus, UK). The limbs were taped to the heat pad. A cervical incision was made at the midline and the muscles retracted to expose the right common carotid artery. The carotid artery was dissected to avoid damage to the vagal nerve and blood vessels. Silk sutures (6-0) were placed around the right common carotid artery and the distal suture at the cranial end suture was firmly tied to allow fixation and manipulation of the vessel. The proximal suture at the caudal end was retracted with haemostats to occlude blood flow during cannulation but not tied. The other two sutures were loosely placed in the middle of the carotid artery to secure the catheter in place after being inserted. A tiny cut was made in the right common carotid artery at the proximal end to allow the insertion of the catheter. The catheter was then inserted into the artery and pushed to the end closest to the junction to the heart and the middle sutures were tied to hold it in place. The caudal end suture was then released to allow the catheter to advance to the heart. The pressure was recorded during the insertion of the catheter and when the diastolic pressure dropped (~80 to ~5 mmHg) it indicated that the catheter had entered the LV. The position of the catheter was then optimised following which baseline measurements were recorded for 10 min in a steady state. The inferior vena cava was then occluded to investigate cardiac function in a load-independent manner. Data were acquired using an acquisition (DAQ) device (PowerLab, ADInstruments) controlled by LabChart (ADInstruments) and analysed by LabChart Pro.

**Organ collection and lung drying**

Tissue weight was measured using an electronic balance (Ohaus Pioneer PX Analytical Balance). The heart was excised from the chest and perfused with 5 mL of saline via the aorta to rinse out blood from the coronary arteries after which it was blotted dry on tissue paper and weighed. The heart was then sectioned under the microscope to obtain the LV, which also was weighed. The lung was collected and cleaned from blood clots, the trachea, and bronchi under the microscope. The wet lung was weighted and then dried at 65°C using Speed-Vac Concentrator (DNA120 OP; ThermoFisher Scientific, UK). Serial weights were taken until dry lung weight remained constant. The dried lung was then weighed, and the wet/dry lung weight was calculated.

**Histology**

Hearts were quickly removed, flushed with saline, and fixed in 10% buffered formalin (Sigma-Aldrich, UK) for 24h before being embedded in a wax block for sectioning. Cardiomyocyte size was assessed by staining adjacent sections from the middle of the LV with AlexaFluor-594 conjugated wheat germ agglutinin (WGA; Invitrogen, UK). Briefly, de-waxed and rehydrated sections were boiled in sodium citrate buffer for 10 min, followed by blocking in 1% BSA/PBS with 5% goat serum for 1h. Sections were then incubated with 10µg/mL WGA for 1h in the dark at room temperature. Sections were mounted in ProLong Gold with DAPI (Invitrogen, UK). Images were analysed for each heart separately by taking 3 fields by Zeiss microscope (ZEISS LSM 900) from 2 different sections for each heart then analysed by ImageJ (50 cells per image/150 per section/300 cells per heart to avoid cell selection bias). Mean cell size and length were determined as the average of all measurements across all 6 images per heart.

**RNAScope**

Dual RNAScope staining (ISH) was performed on 4µm formalin fixed paraffin embedded sections which had previously been incubated at 60⁰C for 2 hours. ISH staining was performed on a Leica Bond Rx auto stainer. Dual ISH detection for Mm-*Runx1* (406678), Mm-*PCM1*-C2 (48688-C2), Duplex control probes (*PPIB*-C1, *Pol2ra*-C2) (320768, Bio-Techne) and *dapβ* (320758, Bio-Techne) mRNA was performed using RNAScope 2.5 LS Duplex reagent kit (red/brown) detection kit (322440; Bio-Techne) strictly according to the manufacturer's instructions. Mm-*Runx1* was stained red and Mm-*PCM1* was stained brown using the duplex kit. Once staining was completed, sections were dried at 60⁰C for 30 minutes and cover-slipped with Ecomount (320409, Bio-Techne). Stained slides were digitally scanned using a Zeiss Axioscan 7 slide scanner with a 20x/0.8NA objective to generate high-resolution whole-slide images for analysis. Heart slice images (6 in each group) were analysed using ZEN Blue software (Zeiss). Eight images in each quadrant were analysed at 60x magnification. Each of the eight images was chosen randomly with the only criteria that each image had at least one nuclei with both positive *PCM-1* (Cardiomyocyte) and *Runx1* staining (*Runx1* positive cardiomyocyte) which was corrected for in the final analysis. Results were presented *PCM-1+* and *Runx1+* cells as a % of *PCM-1+* cells.

**RNA sequencing sample preparation and analysis**

RNA was extracted using small [<200 nt] and large [>200 nt] nucleotide separation with the miRNeasy Mini Kit (Qiagen, UK) from LV myocardial tissue from Day 0 CTRL-*Runx*1^fl/fl^ and CTRL-*Runx*1^Δ/Δ^ mice and Week 13 2HM-*Runx*1^fl/fl^ and 2HM-*Runx*1*^Δ/Δ^* mice.

**Generation of cardiomyocyte-specific Runx1 deficient HFpEF mice**

*Runx*1^Δ/Δ^ mice were generated as previously described^3^. Mice were fed a 60% Kcal/100 g high-fat diet and were given L-NAME in drinking water at a concentration of 1 g.L^-1^ for the first 2 weeks, followed by 1.5 g.L^-1^ for the 3^rd^ and 4^th^ weeks then 1.75 g.L^-1^ until the end of the experiment at 13 weeks (weeks 5-13). Control mice received tap water and were fed a standard chow diet with 7.37 % Kcal/100 g. Body weight and blood pressure were assessed weekly. Endpoint exercise testing, echocardiography and PV loop were conducted and then mice were humanly killed, and organ weights were collected. All transgenic mice were genotyped using rtPCR by *Transnetyx* for presence of the Cre and floxed alleles.

**AAV9-mediated knockdown of Runx1** **in male mice**

12-week-old male C57BL/6N mice were intravenously injected via the tail vein with either AAV9-scramble-shRNA or AAV9-*Runx1*-shRNA (1x10^10^ virus genomes per mouse). To ensure consistent viral titer, single-use aliquots were prepared for each cohort to prevent freeze-thaw cycles. Subsequently, both groups (knockdown and Scramble) were fed a high-fat diet and L-NAME in drinking water at a concentration of 0.5 g.L^-1^ from the start until the end of week 8. Weekly assessments of blood pressure, body weight, and echocardiography were conducted. In addition, endpoint PV loop surgery was conducted, and organ weights were collected.

**Ro5-3335 inhibition of RUNX1**

Ro5-3335 (Tocris-Bioscience, UK) was prepared in 100% DMSO (Fisher Scientific, UK) to a concentration of 100 mM under sterile conditions. The dosage of Ro5-3335 was calculated based on the average weight of mice to be injected in each cohort. For the control mice, an equivalent volume of 100% DMSO was prepared. C57BL/6N mice were given 0.5 g.L^-1^ L-NAME in drinking water and fed a high fat diet for 10-12 weeks. Prior to treatment (typically at weeks 10-12) diastolic dysfunction was confirmed by measuring E/A ratio using echocardiography. Mice exhibiting diastolic dysfunction were selected for the study, while those without diastolic dysfunction were excluded. Mice with diastolic dysfunction underwent exercise intolerance testing and blood pressure measurement. Mice were then allocated to two equal groups (small molecule inhibitor *vs* control) according to their pre-injection E/A ratio, exercise intolerance testing, and blood pressure measurements. The first group received 20 mg/kg Ro5-3335 via subcutaneous injections every other day for 2 weeks, and the second group received equivalent volume of control vehicle DMSO. Post-treatment (endpoint) measurements were taken including echocardiography to assess wall thickness and E/A ratio, exercise intolerance testing, and blood pressure. Animals then underwent PV loop surgery and were sacrificed, and organ weights were collected. The Ro5-3335 study was conducted in a blinded fashion, by which the person conducting the study was blinded to treatment during data collection and analysis.

**AAV9-mediated knockdown of Runx1 in female mice**

14-week-old female C57BL/6N mice were fed with an HFD and g.L^-1^ L-NAME for 4 weeks. Subsequently, the concentration of L-NAME administered was increased to 1 g.L^-1^ for additional 2 weeks, further increased to 1.5 g.L^-1^ for another 2 weeks, and finally increased to 1.75 g.L^-1^ until the conclusion of the study. At week 9, diastolic dysfunction was confirmed through echocardiography by assessing the E/A ratio. Mice demonstrating diastolic dysfunction were included in the study, while those without such dysfunction were excluded. Subsequently, mice were divided into two equal groups (AAV9-scramble-shRNA vs AAV9-*Runx1*-shRNA) based on their pre-injection E/A ratio, results from exercise intolerance testing, and blood pressure measurements. Each mouse received an intravenous injection via the tail vein of either AAV9-scramble-shRNA or AAV9-*Runx1*-shRNA (1x10^11^ virus genomes per mouse). Following injection, measurements were taken at the endpoint, including echocardiography to assess wall thickness and E/A ratio, exercise intolerance testing, and blood pressure evaluation. Subsequently, animals underwent PV loop surgery, were then sacrificed, and organ weights were collected. The female AAV study was conducted in a blinded manner, wherein the individual conducting the study remained unaware of the treatment during data collection and analysis.

**Large Scale Analysis of Human cardiac RUNX1 expression profiles**

The LandExplorer function within Ingenuity Pathway Analysis (Human B38_GC33) was used to explore more than 700,000 curated gene expression profiles^4^. Human RUNX1 expression was explored using the human disease portal comparing disease versus control experiments. Significantly differentially expression of human *RUNX1* (p<0.05) was identified, and datasets were further filtered to identify human cardiovascular disease and cardiac tissue (atria and ventricles) identifying human cardiac Runx1 differential expression in several cardiac disease areas in 11 studies; myocardial infarction (4 studies), hypertrophic cardiomyopathy (3 studies) and dilated cardiomyopathy (4 studies). In each study human RUNX1 expression was significantly increased (Supplementary Figure 5, Supplementary Table 3).

**References**

1. Schiattarella GG, Altamirano F, Tong D, French KM, Villalobos E, Kim SY, et al. Nitrosative stress drives heart failure with preserved ejection fraction. Nature. 2019 Apr 18;568(7752):351–6.
2. Lim GB. New mouse model reveals nitrosative stress as a novel driver of HFpEF. Vol. 16, Nature Reviews Cardiology. Nature Publishing Group; 2019. p. 383.
3. McCarroll CS, He W, Foote K, Bradley A, McGlynn K, Vidler F, et al. Runx1 deficiency protects against adverse cardiac remodeling after myocardial infarction. Circulation. 2018;137(1):57–70.
4. **Qiagen IPA LandExplorer**; https://digitalinsights.qiagen.com/products-overview/discovery-insights-portfolio/analysis-and-visualization/qiagen-ipa/
